# THE IMPACT OF CARBON TAXATION AND REVENUE RECYCLING ON U.S. INDUSTRIES*

**DOI:** 10.1142/S2010007818400055

**Published:** 2018

**Authors:** NICK MACALUSO, SUGANDHA TULADHAR, JARED WOOLLACOTT, JAMES R. MCFARLAND, JARED CREASON, JEFFERSON COLE

**Affiliations:** Environment and Climate Change Canada, 200 Boulevard Sacré-Coeur, Gatineau, Québec, K1A 0H3, Canada; NERA Economic Consulting, 1255 23rd Street, NW, Suite 600, Washington, DC 20037, USA; RTI International, 3040 E. Cornwallis Rd., Durham, NC 27709, USA; U.S. Environmental Protection Agency, 1200 Pennsylvania Avenue NW, Washington, DC 20460, USA; U.S. Environmental Protection Agency, 1200 Pennsylvania Avenue NW, Washington, DC 20460, USA; U.S. Environmental Protection Agency, 1200 Pennsylvania Avenue NW, Washington, DC 20460, USA

**Keywords:** Climate change, carbon tax, economic modeling, inter-model comparison, revenue recycling, energy

## Abstract

This paper provides a detailed, cross-model analysis and discussion of the implications of carbon tax scenarios on changes in sectoral output, energy production and consumption and the competitiveness of the United States’ economy. Our analysis focuses on the broad patterns apparent across models in both qualitative and quantitative terms at the sector level, with a focus on energy-intensive, trade-exposed sectors. We identify how variations in carbon tax trajectories and different options for using the revenue from the tax drive these results.

## 1. Introduction

It is a generally accepted fact among economists and scholars that a properly implemented carbon tax is the most economically efficient way to reduce a country’s carbon emissions. It can also be an effective and efficient way to prompt innovative economic activity (e.g., Pearce, 1991; Fischer and Newell, 2008; Goulder and Hafstead, 2018; Kaufman et al., 2016; Williams, 2016). In February 2017, the Climate Leadership Council (CLC) released a white paper advocating for a carbon tax as a pro-growth policy that would rebalance trade, promote jobs, and help working class Americans (Baker III *et al.,* 2017).^1^ Shortly following the release of this white paper, Senators Sheldon Whitehouse (D-RI) and Brian Schatz (D-HI), as well as Congressmen Earl Blumenauer (D-OR) and David Cicilline (D-RI) proposed legislation in July 2017 entitled the American Opportunity Carbon Fee Act.^2^ This proposed legislation highlights the renewed interest in the United States (U.S.) for carbon pricing and appears against the backdrop of increased international acceptance of carbon pricing policies.

To address climate change, countries adopted the Paris Agreement at the Conference of the Parties meeting (COP21) in Paris on 12 December 2015. The Agreement entered into force less than a year later. Under the agreement, countries set their own targets for reducing emissions of carbon dioxide and other greenhouse gases (GHGs), with a goal of keeping global warming below 2°C compared with preindustrial times (and an aspirational goal of 1.5°). The targets are not legally binding but countries must update them every five years. Carbon pricing is one of the policy instruments that appear in the submitted National Determined Contributions (NDCs) which outline a country’s goal for national effort to address emissions (Environmental Defense Fund and International Emissions Trading Association, 2016). As of 2017, over 40 Nationally and 25 subnational jurisdictions are using some form of carbon pricing such as an emissions trading system or a carbon tax as a way of regulating GHG emissions. Together, these jurisdictions are responsible for emitting approximately one-quarter of global GHG emissions (see Figure S1 in the Supplementary Material).^3^ In North America, Mexico implemented a carbon tax on 1 January 2014 and is currently planning for the launch of an emissions trading system (ETS) in 2018. In October 2016, Canada announced that all provinces and territories must have a price on carbon by 2018 and that each provincial approach must adhere to a set of minimum standards (Trudeau, 2016; Environment and Climate Change Canada, 2017a; 2017b). Furthermore, various carbon tax and emissions trading systems already exist across a series of subnational regions in North America (World Bank and Ecofys, 2017; Taraska and Marano, 2016). This paper explores the impact of carbon pricing in the U.S.

The debate over whether the U.S. should implement a carbon tax is both important and controversial. Supporters of a carbon tax argue that it is the most efficient way of addressing climate change and can support broader fiscal reform. If implemented properly, a carbon tax helps consumers and firms make more economically efficient decisions regarding their use of fossil fuels and leads consumers and firms to shift toward using cleaner fuels including renewable energy sources. Moreover, it spurs the market deployment of innovative and efficient technologies and processes that ultimately enhance energy efficiency and U.S. competitiveness. Supporters further argue that any negative impacts on U.S. competitiveness could be mitigated by a border carbon adjustment (BCA) for goods imported from or exported to countries without carbon policies.

Opponents of a carbon tax suggest it harms the economy by increasing energy costs and has almost no effect on the climate if the U.S. were to impose a carbon tax unilaterally. In the absence of any mitigating measures by its trading partners, the adoption of a nation-wide carbon tax could place energy-intensive industries at a disadvantage relative to international competitors who do not face a similar carbon pricing policy. Therefore, countries with a carbon price could be further disadvantaged in global trade if other large economies do not follow suit.

The introduction of carbon pricing on carbon-dioxide-equivalent (CO_2_e) emissions would likely lead some manufacturing activity and its associated emissions to shift to countries that do not yet have comparable GHG regulations (Böhringer *et al*., 2015; Hoen et al., 2015). The resulting “emissions leakage” would in turn undermine the environmental effectiveness of U.S. action to reduce GHGs. This “leakage issue” was first recognized by the drafters of the American Clean Energy and Security Act of 2009 (H.R. 2454), commonly known as the Waxman-Markey Bill, which established criteria for identifying energy-intensive trade-exposed industries.^4^ Specifically, H.R. 2454 considers an industry to be “presumptively eligible” for emission allowance allocations (or “rebates”) if the “trade-vulnerable” industry’s energy intensity or GHG intensity is at least 5%, while its trade intensity is at least 15%. H.R. 2454 considers an industry to be “presumptively eligible” if its energy or GHG intensity is at least 20%, regardless of its trade intensity.

The implication of introducing a U.S. carbon price is explored in the Stanford Energy Modeling Forum Model Inter-comparison Project number 32 (EMF 32) entitled “*The Study on U.S. Carbon Tax Strategies*”. EMF 32 focuses on the development and cross model comparison of results from U.S. climate policy scenarios focusing on different carbon tax trajectories and different options for using the revenues from the tax. The scenarios did not impose any border carbon adjustments (BCAs). This paper uses results from EMF 32 to explore the broad implications of carbon pricing across the U.S. economy.

The remainder of this paper is organized as follows: Section 2 describes the basic methodology and approach that guided the EMF 32 modeling and cross model comparison. It highlights the key attributes of the models participating in the study, as well as the key assumptions and approaches. Section 3 presents the study’s main results as they pertain to the impact of carbon taxes on changes in sectoral output, energy production and consumption, and the competitiveness of the U.S. economy. Section 4 offers some overall conclusions and policy implications.

## 2. Methodology

To varying degrees, each sector of the economy emits carbon dioxide, and all sectors of the economy are economically inter-linked. An economy-wide carbon tax directly affects each sector in ways that are approximated by straightforward applications of economic theory. Much less straightforward are the indirect consequences of the policy, such as how increasing fuel prices — transmitting through the numerous linkages among sectors — drives supply and demand for goods and factors of production, trade in goods and services, along with household incomes and expenditures. Economy-wide models help us trace the path of complex economic forces, and offer policy insights about indirect effects that may be significant and often counterintuitive. The results examined here illustrate how the mix of economic forces, captured in these economy-wide models, lead to changes in sectoral outcomes under the various carbon tax and recycling scenarios.

While there are limitations with economy-wide models, these models offer us a useful policy analysis tool. They rely on stylized representations of a future economic trajectory with foreseeable changes in policies, as well as resource and technology endowments. Yet many of these policy changes are unforeseeable. Most often, the forecasted economic outlook these models use as their baseline scenario will likely not match the trajectory the economy takes in the future. Despite this, economy-wide models can still illuminate the manner and extent to which a policy induces changes in economic outcomes and trade-offs relative to a future economic trajectory.

Eleven models were included in the carbon tax strategies study. Although 10 of the 11 models are computable general equilibrium economic models, the models differ in a number of ways that may have implications for results. An overview of the models participating in this study is available in McFarland et al. (2018). Because this paper examines the sectoral and energy outcomes, additional details on the sectoral composition may be found in Table 1. These models represent several mechanisms by which the economy might accommodate a carbon tax. Trade patterns might shift such that industries and consumers buy their goods from regions that produce goods at a relatively lower price, either due to their processes being less carbon intensive or because they come from jurisdictions where they face a smaller or no carbon tax.

Producers and consumers may substitute carbon-intensive goods for less carbon-intensive goods (energy goods or otherwise). Producers and consumers may buy fewer goods all together under a carbon constrained regime. The combination of these substitutions (e.g., reallocation of capital and labor and expenditure behaviors) toward less carbon-intensive energy sources and production technologies and less economic activity will drive the overall level of abatement observed. In general, we expect those models that represent greater flexibility in substitution behavior and a wider set of low-carbon technologies (e.g., USREP-ReEDS, NEMS) will report larger levels of abatement for a given tax, all else being equal.

This study examines potential implications of an economy-wide carbon pricing policy by varying two key parameters: the trajectory of the carbon price and the use of the revenue. All the scenarios apply the carbon tax to all fossil fuel CO_2_ emissions, which represent roughly 77% of overall gross U.S. GHG emissions (EPA, 2017). The results presented in this paper focuses on four “core” price trajectories in which the tax begins in 2020 at either $25 or $50 and rises at either 1% or 5% per year (see Table 2). In conjunction with these fixed price trajectories, this paper reports results for “revenueneutral” revenue recycling. In all scenarios, government spending is held at baseline levels and net revenues are returned to households either lump-sum or via cuts in the marginal tax rates on capital or labor income.

The results presented here depict the market outcomes simulated by the economy-wide models analyzed.^5^ This focus on market outcomes carries an implicit bias toward policy costs, as households do not make market purchases of the environmental benefits that motivate the policy. Many models track the changes in emissions that give rise to these environmental benefits; however, assigning an economic value to those emissions reductions is a separate exercise not undertaken here (for example West et al., 2013; Woollacott, 2018). A complete analysis of carbon policies would require a comparison of its costs and benefits. Such an assessment is beyond the scope of this work, but understanding the sectoral outcomes of the policy remains a valuable component to understanding its costs.

## 3. Results

### 3.1. Energy use

Putting a price on carbon emissions is intended to reduce the demand for fossil fuel-based energy sources and encourage the use of low or nonemitting energy sources such as natural gas, renewables (e.g., hydro, wind, solar, etc.) and nuclear. A carbon tax increases the cost of fossil fuels that emit more carbon encouraging consumers and businesses to switch to other, often cleaner fuels. Although these fuels may be initially more expensive, the higher price induces users to use lower-carbon energy sources, reduce energy use, or use energy more efficiently. The EMF 32 modeling and cross model comparison results are consistent with this theory. Modeling results clearly show that implementing a carbon tax lowers the demand for emissions-intensive energy sources such as coal and oil, while it increases the demand for relatively less emissions-intensive energy sources such as natural gas and renewables.

For EMF 32, the U.S. Energy Information Administration’s (EIA) Annual Energy Outlook 2016 reference scenario excluding the Clean Power Plan guides the baseline behavior for almost all models.^6^ Despite this harmonization, there is variation in primary energy use across the models. In addition, four models (DIEM, G-Cubed, IGEM, and ADAGE-US) focus on fossil fuels and do not explicitly account for renewables in the model. This could have consequences on compliance costs.

**Figure 1.**
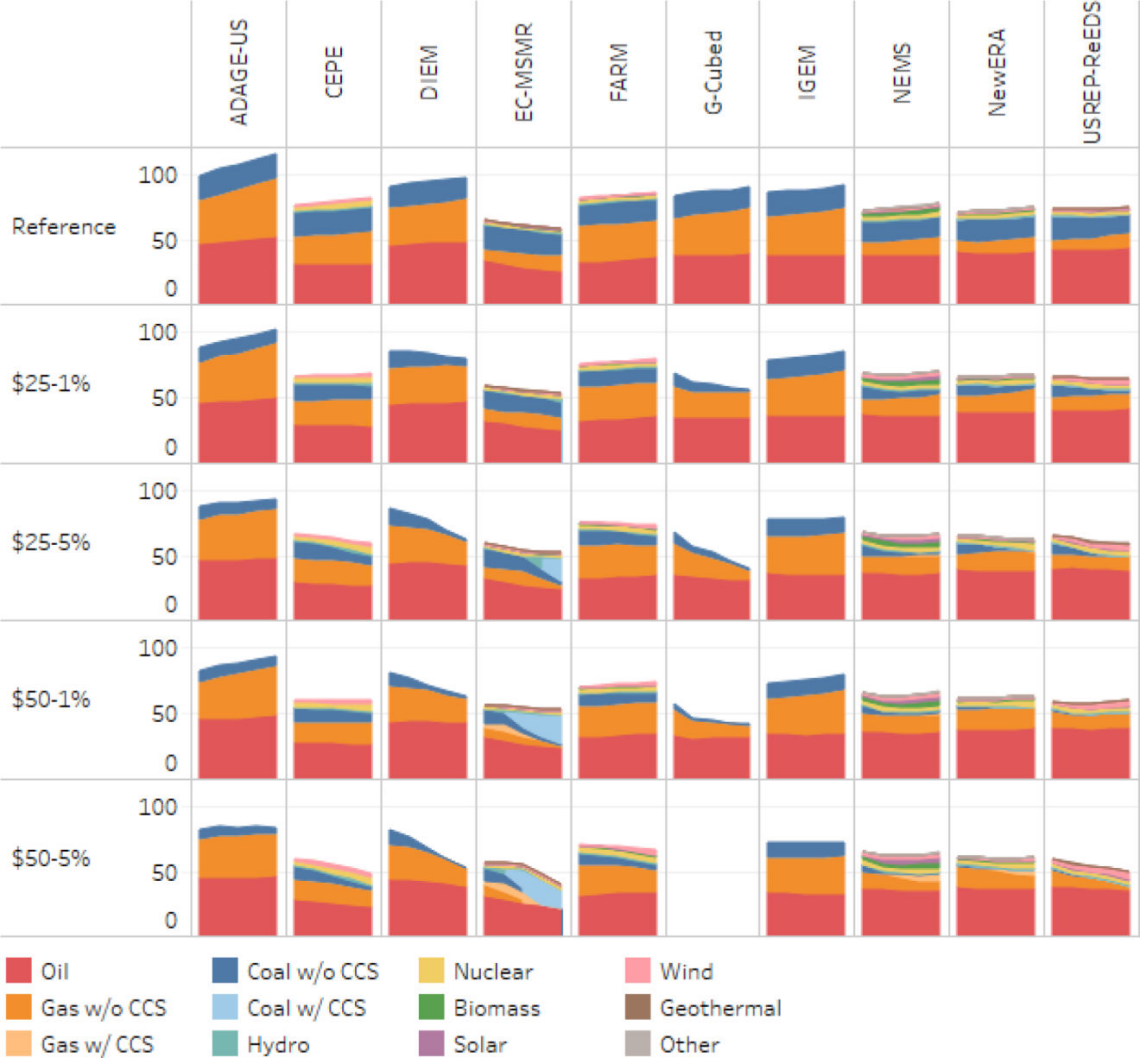
Impact of carbon pricing on primary energy demand (exajoules per year).

Figure 1 depicts primary energy demand by fuel and sector under the reference case and the carbon tax scenario for the 10 participating modeling teams (i.e., ADAGE-US, CEPE, DIEM, EC-MSMR, FARM, G-Cube, IGEM, NEMS, N_ew_ERA, and USREP-ReEDS). With respect to the carbon tax scenarios, the figure depicts results for the following four carbon tax trajectories under the lump-sum rebate to households revenue recycling option: (i) $25/tonne increasing at 1%/year; (ii) $25/tonne increasing at 5%/year; (iii) $50/tonne increasing at 1%/year; and (iv) $50/tonne increasing at 5%/year.

The EMF 32 cross-model comparison shows that without any new carbon taxes primary energy steadily increases during the period 2020–2040 to support economic growth. Total primary energy usage grows at an average annual rate of 0.3% per year to reach 98 exajoules by 2040.^7^ From 2020 to 2040, this represents a cumulative increase of 7%. Of this total, −5.0% comes from coal (17 exajoules), 19% from natural gas (32 exajoules), 2% from oil (39 exajoules), and 20% (10 exajoules) from nonfossil fuels, which includes hydropower, nuclear, and renewable fuels. The fuel-specific values vary across the models.^8^

Focusing on the model specific change in primary energy between 2020 and 2040, seven of the models (CEPE, DIEM, FARM, G-Cubed, IGEM, NEMS and N_ew_ERA) assumed that primary energy would increase in the range of 0.3% to 0.4% per year, while USREP-ReEDS assumed a growth of 0.02%. The EC-MSMR suggests a decline of 0.5% per year. Unlike the other models, whose reference cases are closely aligned to the AEO2016 forecast, the EC-MSMR reference case is aligned to a version of the AEO2015 (which includes the impacts of the Clean Power Plan as reported in the 2016 Second Biennial Report of the United States of America^9^).

Each model, except EC-MSMR, shows a consistent pattern of overall growth in primary energy use, although the growth rates of the specific primary fuels vary. Four of the models (DIEM, G-Cubed, IGEM, and ADAGE-US) do not report nonfossil fuel primary energy sources. By 2040, these models suggest that the share of coal and oil (in total primary energy use) declines, while the share of natural gas increases. USREP-ReEDS projects the greatest decline in coal’s share of total primary demand (−5.2%), followed by the ADAGE-US (−2.7%), while ADAGE-US and IGEM project the greatest decline in the share of oil (−3.2%) followed by G-Cubed (−2.6%). With respect to natural gas, ADAGE-US (5.8%) and IGEM/G-Cubed (5.1%) project an increase in its share.

There is also variation among the models that include both fossil fuels and renewables. For example, EC-MSMR (0.3%) projects an increase in coal’s share of total primary energy, while the other models show decreases ranging from FARM 3.1 (1.1%) and USREP-ReEDS (5.2%). While most models show a decline in oil’s share, two show an increase — FARM 3.1 (2.6%) and USREP-ReEDS (2.0%). With respect to hydro, CEPE projects an increasing share (0.5%), while FARM 3.1 suggests a decreasing share (−0.1%). NEMS projects the greatest increase in solar (1.0%). There is also variation in the changing shares of wind and biomass.

Figure 1 also shows that implementing a carbon tax will lower the demand for emission intensive energy sources such as coal and oil, while increasing the demand for energy sources such as natural gas and renewables. This is consistent with other studies (e.g., Hafstead and Picciano, 2017; Aldy, 2013). In general, $50/tonne would add about 44 cents to a gallon of gasoline, 51 cents to a gallon of diesel, $2.65 per MMBtu of natural gas, while coal prices would increase between $63 and $129 per tonne depending on the type of coal being used.^10^

The cross-model comparison shows that as the carbon price increases, the level of primary energy usage, especially the use of coal, declines. One exception is the EC-MSMR model, which shows under moderately increasing carbon prices, integrated gasification combined cycle (IGCC) with capture is assumed to be competitive and this leads to an increase in coal use. The fuel share mix is in general consistent across the models; however, the magnitude of the impact increases with the increasing carbon prices. For example, average total primary demand across the models in 2040 is 67 exajoules ($50/tonne increasing at 5%/year scenario). This compares to 76 exajoules ($50/tonne increasing at 1%/year scenario). The $25/tonne scenario exhibits the same general pattern.

Focusing on specific model results, total primary energy demand ranges from 84 exajoules (ADAGE-US) to 48 exajoules (CEPE) under the $50/tonne increasing at 5%/year scenario, while it ranges from 94 exajoules (ADAGE-US) to 60 exajoules (CEPE) under the $50/tonne increasing at 1%/year scenario. The model results also suggest the importance of the availability of nonfossil fuels in the mix. The models that do not have the option to substitute to nonfossil fuels tend to have results showing larger net reductions in total primary energy use. This is mostly due to significant demand reduction for fossil fuels.

**Figure 2.**
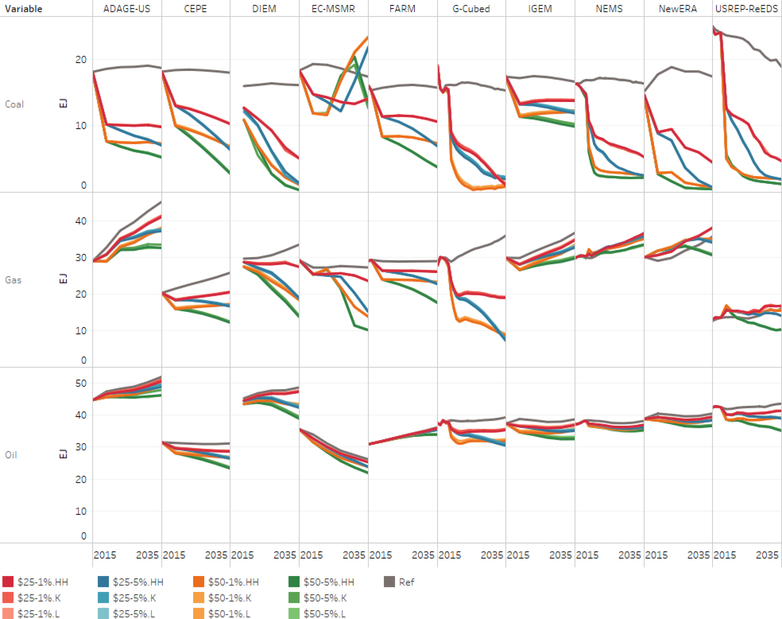
Change in primary energy demand from lump-sum rebate for capital and labor tax reductions, 2020–2040 (exajoules per year).

While the results clearly show that the carbon tax level does affect total primary energy usage, revenue recycling for each carbon level does not show the same influence. That is, different revenue recycling methods tend to generate similar levels of primary energy use for a given tax rate. This is consistent with other studies (e.g., Ecofiscal Commission, 2016). Figure 2 depicts change in primary energy demand for the various tax level and revenue recycling combinations.^11^ Revenue returned to households either as direct rebates (also called lump-sum transfers) designated by “HH”, while “K” designates cuts in the marginal tax rates on capital and “L” labor income.

As illustrated in Fig. 2, all models suggested that the tax level could reduce primary fossil energy demand in aggregate. With respect to coal, all but one model (i.e., EC-MSMR) suggest that primary coal demand under the “core” carbon tax scenarios will be lower than the reference level demand. The results from EC-MSMR are influenced by the fact that coal with CCS is competitive in the carbon tax scenarios and as such, there is a switch from coal without CCS to coal with CCS. With respect to primary oil demand, virtually all models suggest that oil demand will be lower than the reference level demand. With respect to natural gas, three models (e.g., N_ew_ERA, NEMS, and USREP-ReDS) suggest that there is a carbon price range where natural gas is higher than the reference case.

In the lump-sum revenue-recycling scenario, the cross-model comparison shows that primary energy demand is significantly lower under the carbon price scenarios compared to the reference case scenario across all models; this demand reduction is also true across all revenue recycling scenarios.^12^ However, modeling results show great variation in the sensitivity of energy demand with respect to the carbon price and growth rate. The G-Cubed and DIEM models show the greatest responsiveness to the carbon price increase (primary energy being 55.7% and 36.2% below their respective reference level in 2040). The CEPE and USREP-ReEDS models are also highly responsive to the carbon price increase (primary energy being 28.2% and 26.3% below their respective reference levels in 2040). The decrease in primary energy demand in the other models ranges from 12.6% (NEMS) to 19% (ADAGE-US).

Focusing on the change in primary energy demand for the capital tax revenue recycling relative to the lump-sum rebate, the ADAGE-US, DIEM and IGEM models show the greatest variation across all the tax rates. With the exception of IGEM, all the models show little variation for the labor tax revenue recycling relative to the lump-sum rebate. In general, the analysis suggests that revenue recycling does not have a major impact on energy use. In terms of production inputs, the first-order impact of capital income recycling would be a trade-off between capital and labor while the second-order impact would be value-added and energy. The trade-off between fuels is less impacted since the fuel trade-offs are influenced much more by a carbon price than through substitution between energy and value-add (general in top-down models) or resource allocation. Hence, we see that the fuel use and carbon emissions are relatively similar across different revenue-recycling methods. In the revenue-recycling scenarios, a carbon tax is effective in reducing emissions, but the negative economic consequences of higher energy prices outweigh the economic benefits of the recycling of carbon tax revenues as they are represented in these models. Since it is the carbon price level that affects energy demand the most, the focus will be on how the carbon tax level influences energy use.

**Figure 3.**
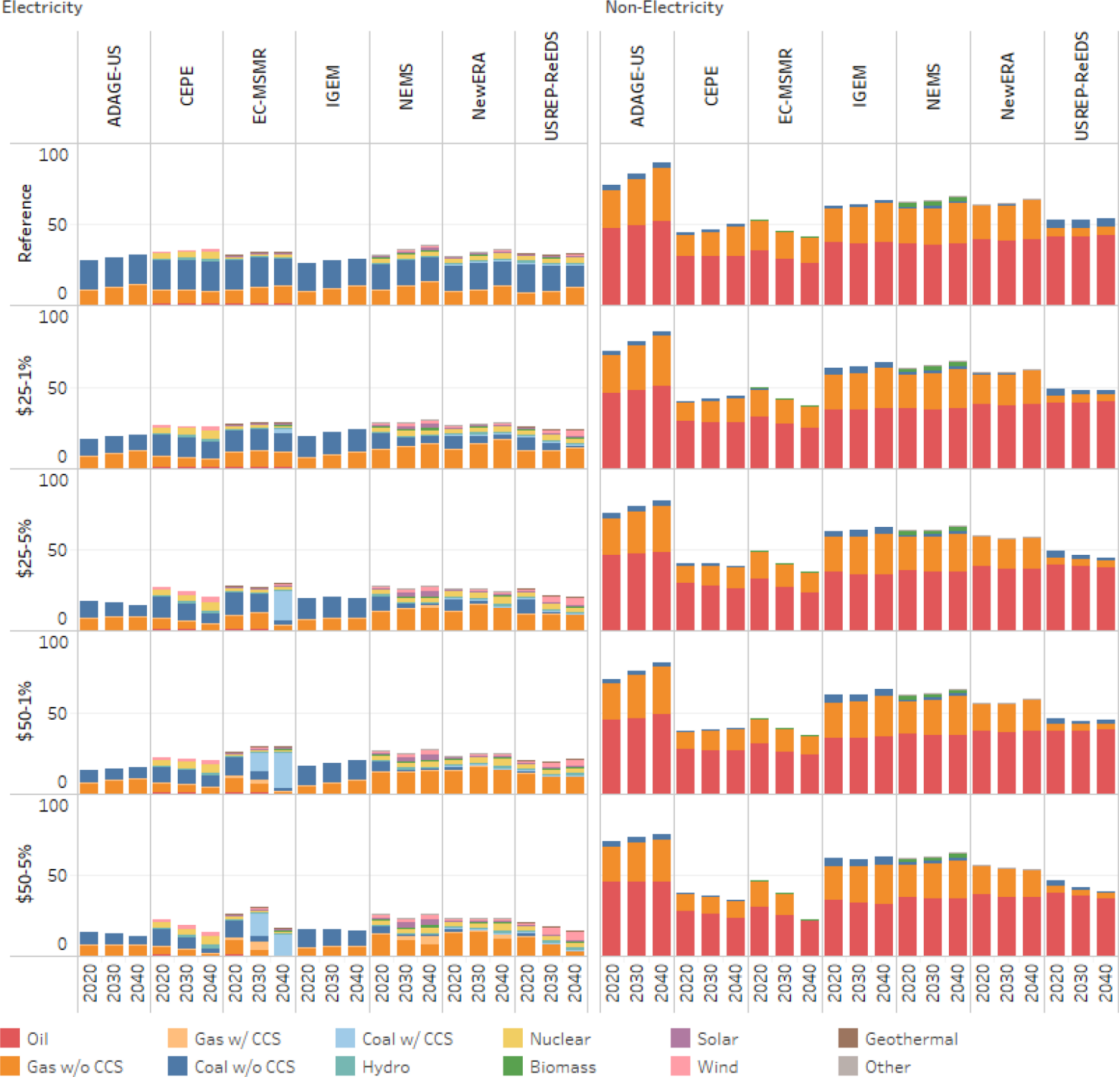
Reference scenario primary energy demand by fuel and sector (exajoules per year).

To better highlight the impact of carbon pricing on renewable technology and the switching from carbon intensive fossil fuels (i.e., coal) to lower carbon-intensive fossil fuels (i.e., oil and natural gas), Fig. 3 depicts primary energy demand by fuel and sector for seven of the participating modeling teams (i.e., CEPE, EC-MSMR, IGEM, NEMS, N_ew_ERA, ADAGE-US, and USREP-ReEDS).^13^ The results are depicted for the reference case and for the carbon tax scenarios. The figure clearly shows the variation in fuel and technology representation across the model. For example, several of the models do not explicitly represent renewable energy sources (e.g., ADAGE-US and IGEM) and this limits substitution toward cleaner fuels, leaving reductions in fossil fuels and/or output as the primary response. For other models such as EC-MSMR, they have access to coal with CSS, and this allows for continued use of coal.

In the reference case, demand for natural gas and oil is highest in the nonelectricity, while the demand for coal and natural gas is highest in the electricity sector. Focusing on the model average for 2020, 88% of coal was used in the electricity sector, while 67% of the natural gas and more than 99% of the oil was used in the nonelectricity sectors. While there was some variation across all the models, USREP-ReEDS showed the greatest variation: the electricity sector consumed 74% of coal and the nonelectricity sectors used 36% of the natural gas.

By 2040, the average across the models projects that the electricity sector will consume 87% of coal, while the nonelectricity sector will consume 64% of the natural gas and some 99% of the oil. The variation in demand is high across the models. With respect to coal use for the electricity sector, the lowest coal use is projected by USREP-ReEDS (75%) and ADAGE-US (80%), with CEPE and IGEM around 90%, while EC-MSMR projects that 95% of coal will be used in the electricity sector.

In the tax scenarios, the figure clearly shows that the use of coal or coal without CCS falls. This fall in coal use is consistent across the models. Focusing on specific models, the IGEM model, while showing a lower demand for coal, continues to suggest a role for coal with CCS (i.e., the blue bar). In EC-MSMR, there is a shift from coal with CCS to coal without CCS over time and because of increasing carbon tax levels.

**Figure 4.**
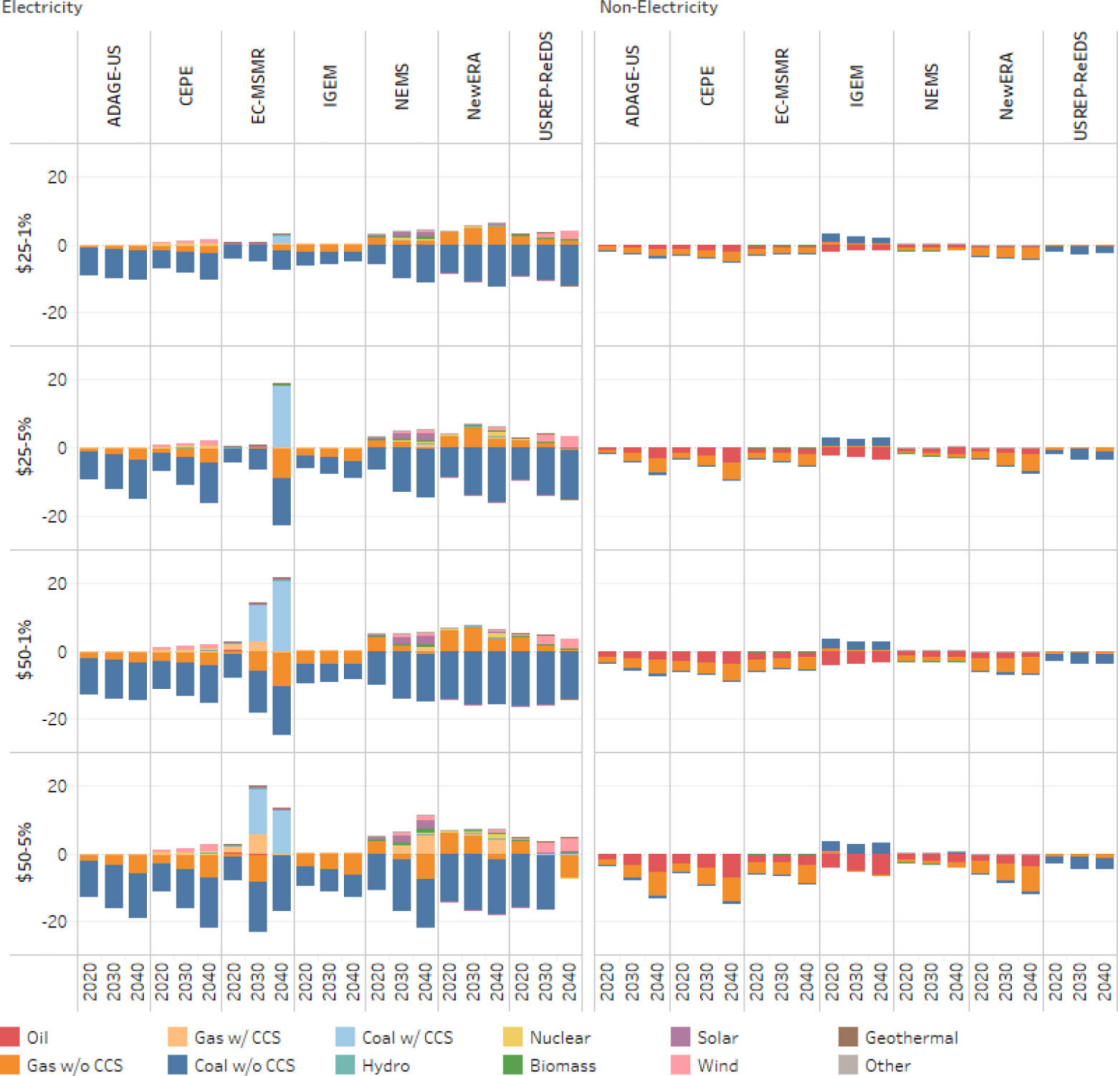
Change in primary energy demand by fuel and sector relative to the reference case (exajoules per year).

Figure 4 depicts the changes in primary energy demand in the electricity sector and nonelectricity sectors for the various carbon price scenarios relative to the reference scenario. The results suggest that the higher the carbon price, the greater the change in energy demand. Figure 4 shows that the carbon tax has a much greater impact on the electricity generation sectors than on the nonelectricity sector. This is in part because the nonelectricity sector (i.e., residential, commercial, transportation and industry) requires much higher carbon prices to induce consumer and producers to invest in energy saving activities or fuel switching. Focusing on the nonelectricity sectors and for the highest carbon tax level (i.e., $50 growing at 5%), on average, the reduction in primary energy demand in 2040 range from 0.40 exajoules for coal, to 3.59 exajoules for natural and 3.70 exajoules for oil. The reductions under the lower carbon price scenarios are lower. For the lowest carbon tax level (i.e., $25 growing at 1%) the reduction in primary energy demand in 2040 range from 0.21 exajoules for coal, to 1.66 exajoules for natural and 1.25 exajoules for oil.

Figure 4 also shows variation across the models. For example, the change in coal demand ranges from 0.34 (EC-MSMR) to 3.49 exajoules (USREP-ReEDS) below the reference levels, to 0.48 (NEMS) to 3.02 exajoules (IGEM) above the reference levels. The cross-model results for natural gas range from 0.59 exajoules below the reference level (IGEM) to 6.88 to 7.42 exajoules below the reference levels from ADAGE-US and USREP-ReEDS, respectively. There is a similar wide range for oil. The results suggest that for the nonelectricity sector the least responsive model is EC-MSMR.

Fuel use in the electricity sector is more responsive to the carbon tax than in other sectors. The various carbon tax levels generate both reductions in fossil-based primary energy and fuel switching from higher carbon intensive fuels to lower carbon intensive fuels and renewables. Focusing on 2040, on average, coal without CCS is some 8.75–11.77 exajoules below the reference level depending on the carbon price level (i.e., $25 growing at 1% compared to $50 growing at 5%). Natural gas without CCS is also lower (i.e., 5.07 exajoules below the reference level for the carbon price scenario at $50 growing at 5%). While the demand coal, natural gas and oil without CCC are lower compared to the reference case, there coal and natural gas with CCS see an increase in demand. The demand for coal with CCS is some 0.38–1.85 exajoules higher than the reference level depending on the carbon price level (i.e., $25 growing at 1% compared to $50 growing at 5%), while natural gas with CCS is some 0.01 to 1.42 exajoules higher than the reference level.

The increased demand for renewable energy sources and nonemitting energy sources is also stimulated by the carbon price levels. For example, wind energy is some 0.59–1.11 exajoules above their reference case levels, while solar (0.22–0.38 exajoules), biomass (0.13–0.26 exajoules), hydro (0.03–0.05 exajoules) and nuclear (0.18–0.90 exajoules) are also higher, on average, than their respective reference levels in 2040.

All models, with the exception of USREP-ReEDS, suggest significant reductions for coal without CCS. For the carbon price scenario of $50 growing at 5%, the EC-MSMR suggests the highest reduction relative to the reference level (16.59 exajoules), followed by N_ew_ERA (15.93 exajoules) with IGEM being the lowest (6.53 exajoules). The reductions for natural gas without CCS range from 1.90 exajoules (N_ew_ERA) to 7.63 exajoules (NEMS) relative to their respective reference levels. EC-MSMR suggests that coal with CCS will be some 12.94 exajoules higher than the reference level, while N_ew_ERA and NEMS suggest a higher demand for natural gas with CCC (i.e., respectively 4.25–5.72 exajoules higher than their respective reference levels). With respect to wind energy, US-REP-ReEDS is the most responsive (4.09 exajoules above the reference level), while NEMS is most responsive for solar (2.31 exajoules above the reference level) and biomass (1.38 exajoules). N_ew_ERA suggests a higher contribution from nuclear (5.52 exajoules above the reference level).

The results underlying Fig. 4 suggest that under the low carbon price scenario, there are more compositional changes taking place with fossil fuels and so the relative demand for renewables is mitigated. However, at a higher annual increase in carbon prices the share of renewables demanded increases while that of fossil fuels decreases (although the magnitude of change is much smaller). This suggests that a steady and higher increase in the carbon price is necessary to expand renewable supply.

From a sector-specific perspective, the results across all models suggest that the electricity sector is more responsive to the carbon price than the other sectors. This is consistent with theory and other findings Paul et al. (2013) and Kaufman et al. (2016) that suggest that the electricity sector has a greater ability to fuel switch and reduce the use of carbon-intensive fuels than do the other sectors (e.g., residential, commercial, industrial, and transportation). In other sectors, demand for less emissions-intensive fuels such as natural gas and biomass increases in both periods in all scenarios.

### 3.2. Changes in household energy consumption and end-use energy prices

**Figure 5.**
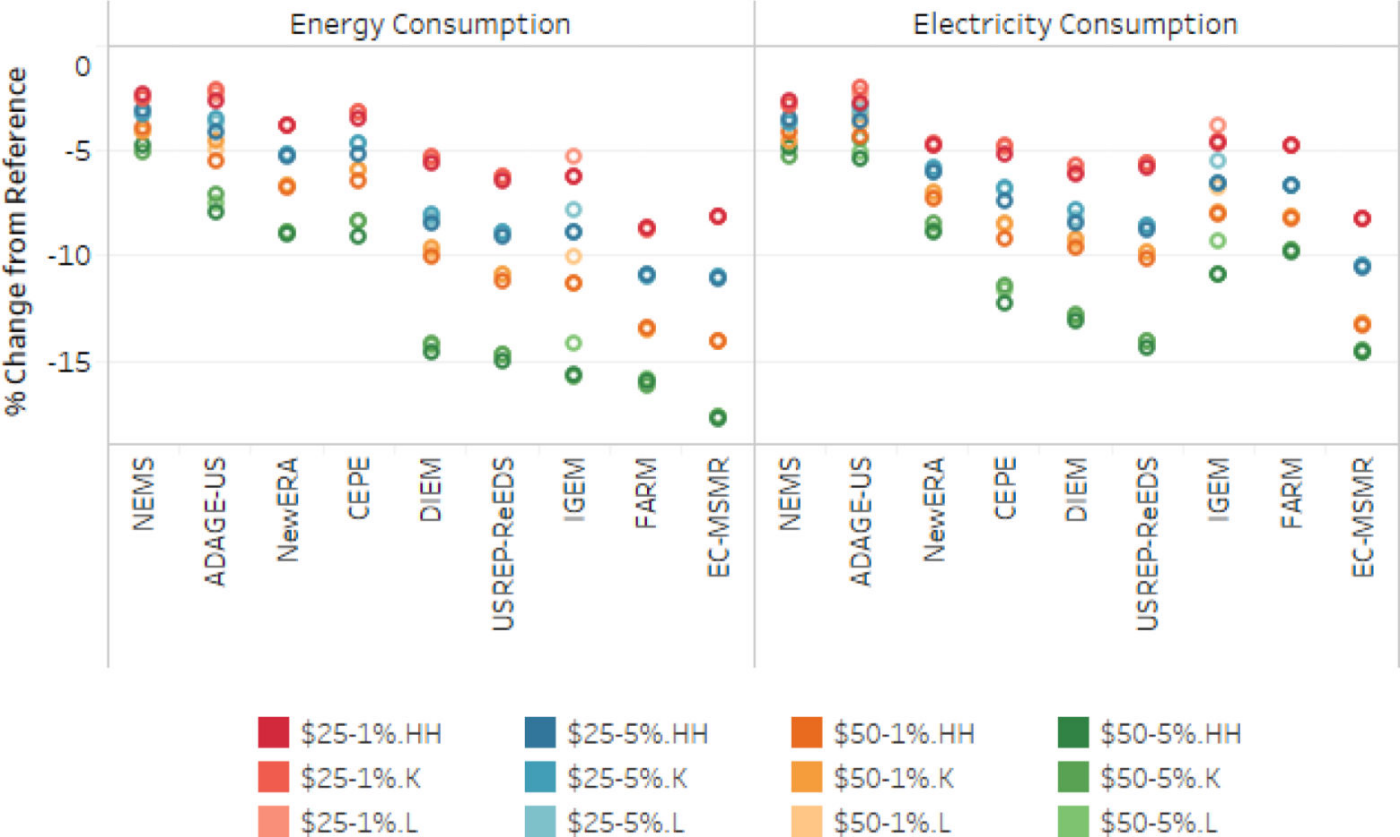
Percent change in energy and electricity consumption (average 2020–2030) by model, tax, and recycling scenario.

This section examines the effect of carbon taxes and revenue recycling on household energy and electricity consumption and end-use energy prices. Figure 5 shows the change in average energy and electricity consumption from 2020 to 2040 across the core tax paths and revenue recycling scenarios (models are sorted by reduction in energy demand in the $50–5 scenario). The decrease in household energy consumption differs substantially across the models. Energy consumption falls by roughly 2%–8% for the $25–1% scenario and 5% to nearly 18% for the $50–5% scenarios. Looking across all tax and revenue recycling options, the results show a bifurcation in response between the top four models (5% decrease in consumption) and the bottom five (11% decrease in consumption). Electricity consumption is slightly less responsive. Though electricity consumption falls by 2–8% for the $25–1% scenario, it falls by 5–15% under the $50–5% scenario. The rank order of the models’ sensitivity to carbon prices is consistent for energy and electricity consumption with the exception of IGEM and FARM, which show lower changes in electricity consumption. The effect of revenue recycling on energy and electricity demand is barely detectable in N_ew_ERA, FARM, and EC-MSMR and between 0.25 and 1.3 percentage points in most other models.

**Figure 6.**
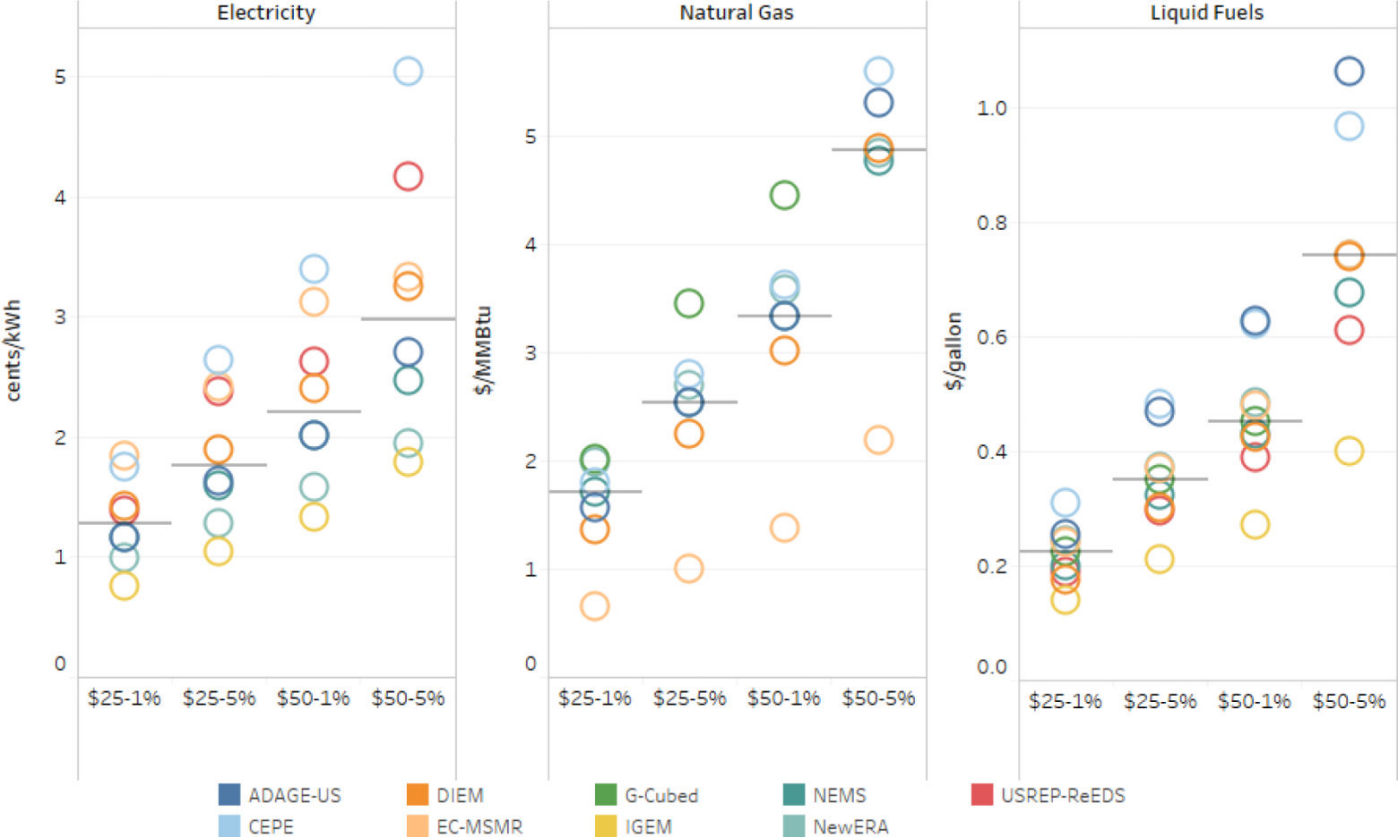
Absolute change in 2020–2040 average end-use energy prices for electricity, natural gas, and liquid fuels by tax scenario with lump-sum recycling. Gray bar is the median.

Carbon pricing of emissions also increases the costs of end-use energy. Figure 6 presents absolute change in average prices from 2020 to 2040 for electricity, natural gas, and liquid transportation fuels across models and pricing scenarios under lump-sum recycling to households. Median price increases across all models and scenarios, the median electricity prices rise by 8–57%; natural gas prices by 12–231%; and liquid fuels by 5–37%.

### 3.3. Change in output

The relative change in output varies significantly by model and scenario. Output changes in scenarios without capital tax recycling are nearly all negative. The lone exception is a slight increase in output reported by the USREP-ReEDS model in the scenarios with labor tax revenue recycling. The pattern of changes was qualitatively similar for the $50–5% scenarios with average magnitudes slightly less than twice as large. In this scenario, the average change in output is several times greater than its $25–5% counterpart.

All models report decreased total output for the economy in the lump-sum scenario ranging approximately 0.5–2.0%. Output changes are larger on average for energy-intensive, trade-exposed (EITE) sectors than for total output, demonstrating their higher than average sensitivity to fuel price shocks from its relatively higher energy intensity.^14^ The IGEM, GH-E3, and NEMS models exhibit some of the largest EITE and total output declines across models. The pattern of total and EITE output declines varied across models. Two thirds of models reported EITE output declines larger than total output declines for the lump-sum scenario with a $25–5% tax. While there is broad agreement that EITE sectors will experience output declines in most cases, models differ as to whether output declines will be particularly large for EITE sectors. All models also reported declines in output for energy-intensive, trade-exposed (EITE) industries in the lump-sum scenario with a range of approximately 0.25–3.75% (see Fig. 9).

Percentage GDP declines were generally smaller than total output declines, indicating that the policy induced the economy to generate more income from a given amount of output (see Fig. 8). The figure depicts a scatter plot of models’ percentage GDP declines (vertical axis) against their total output declines (horizontal axis) under three different policies. In all but one case the GDP decline is smaller in percentage terms than the output decline. The diagonal line indicates where the percentage declines are equal.

**Figure 7.**
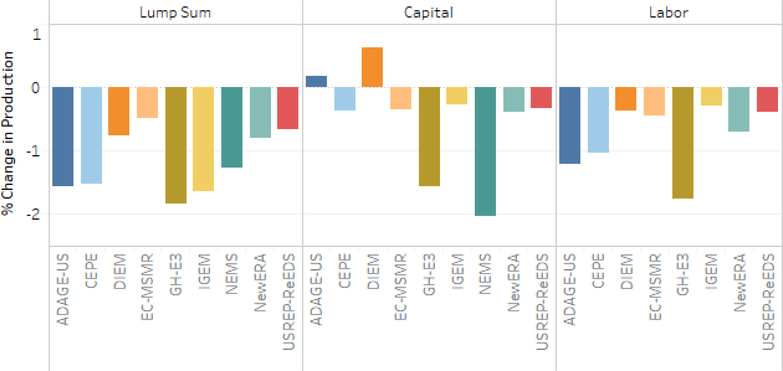
Percent change in total output for $25 at 5% Scenarios, cumulative 2020–2040 discounted at 3%.

**Figure 8.**
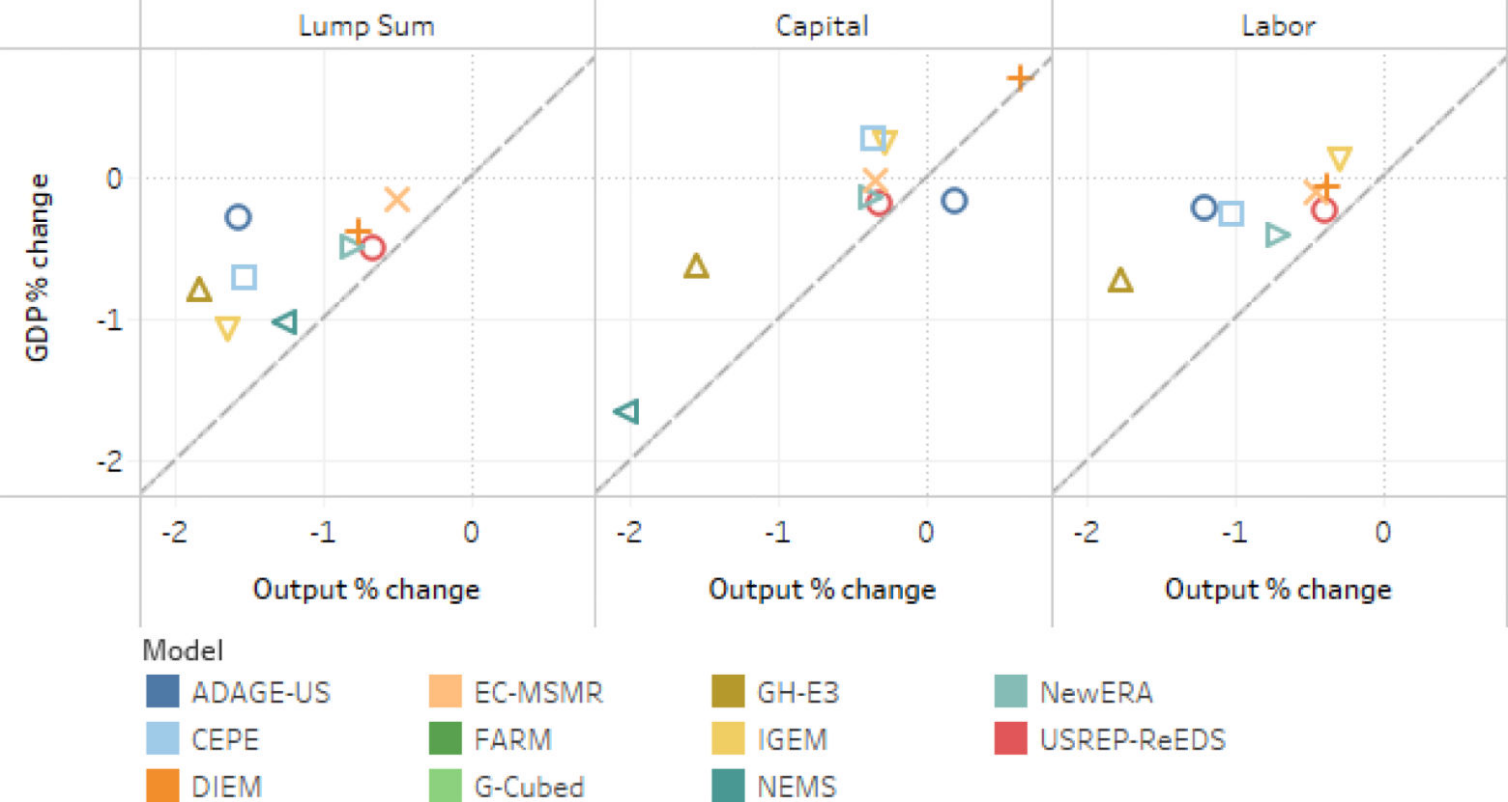
Percent change in the level of GDP versus output relative to baseline for $25 at 5% scenarios, 2020–2040 NPV discounted at 3%.

**Figure 9.**
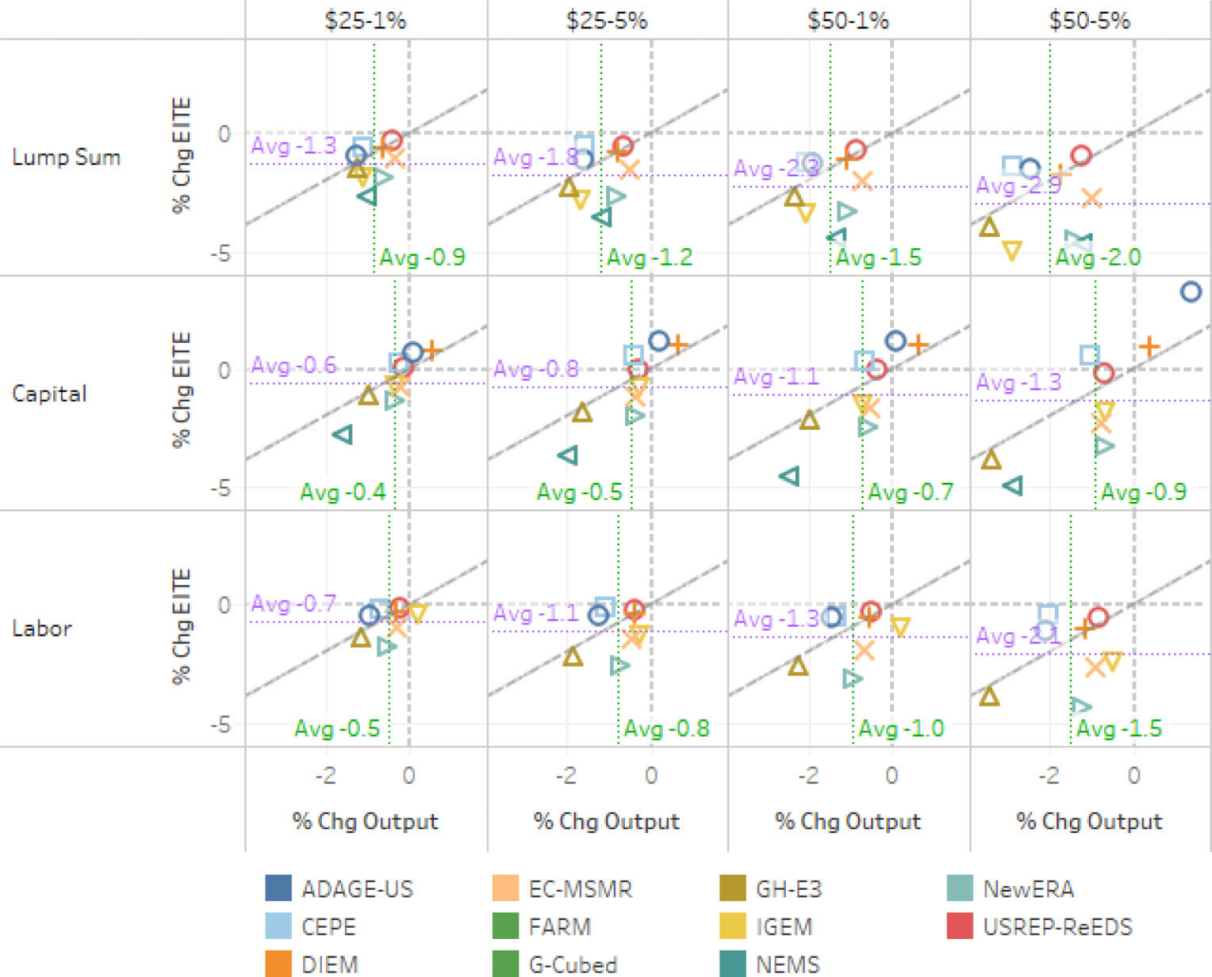
Percent change in EITE output versus total output (cumulative 2020–2040 discounted at 3%). Average change in EITE is in purple and average change in total output is in green. Diagonal line represents equal changes in total and EITE output.

In the capital tax recycling scenario two of the eight models (DIEM and ADAGE-US) report increased total and EITE output and CEPE reports higher EITE output alone (see Figs. 7 and 9). Taxes induce inefficiencies in markets with an associated excess economic burden over and above the revenue they raise. When policies swap taxes with relatively high excess burden (e.g., taxes on factors of production) for taxes with relatively low excess burden, theory suggests the possibility for a “double dividend” where economic activity (typically GDP) can increase because of the change in tax policy (cf. Goulder, 1995).

There is broad model agreement on higher total output in the capital tax-recycling scenario relative to the lump-sum recycling scenario. Results are again mixed as to whether EITE sectors’ output experiences smaller percentage declines than total output under the capital scenario. Models vary in how capital taxes are represented. For those representing only corporate income taxes as capital taxes in their model, the efficiency gains from capital tax recycling could be larger as the tax is applied to a narrower base, providing a larger reduction in the rates, and are likely to be more distortionary than labor income taxes.

Results from the labor tax recycling scenario exhibited a pattern largely similar to the lump-sum scenario, though muted. The reduced impact on total output was most dramatic for IGEM, going from a 1.7% decline to 0.3%. The similarity in pattern with smaller percentage declines was largely true for EITE sectors as well. The mixed scenario with capital tax and lump-sum recycling (not shown) conveys a similar pattern for total output to the lump-sum scenario, though with smaller impacts. Three of five models reporting for this scenario indicate significantly small decreases for EITE sectors, likely a result of the implications of capital tax recycling.

Figure 9 illustrates the relationship between the changes in total output and the changes in EITE output across the core tax and recycling scenarios. The capital recycling scenarios have the lowest average declines in EITE and total output. EITE industries’ output declines relatively more than economy-wide output. Energy price increases have a larger impact on output prices for energy intensive industries and trade exposure enables demand substitution away from domestic production. The average drop in EITE output across the models ranges from 0.2% to 0.9% more than the loss in total output. Higher tax and tax escalation rates lead to lower output and a greater disparity between the total output and EITE output. Recycling carbon tax revenue to offset distorting capital and labor taxes lessens the output loss. Using carbon tax revenue to offset capital taxes is more effective at stemming EITE and total output losses than labor tax or lump-sum recycling. Recycling revenue to offset capital taxes increases EITE production in three models (ADAGE-US, CEPE, and DIEM).

**Figure 10.**
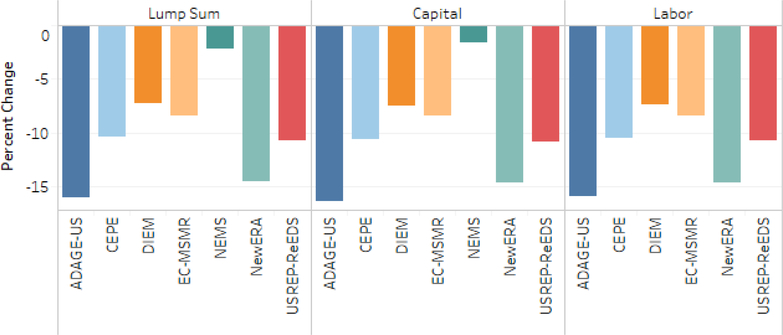
Percent change in industrial energy intensity versus reference for $25 at 5% Scenarios, 2020–2040.

Last, we examine declines in the energy intensity of production. Figure 10 reports the percentage changes for the ratio of final energy to total output. This provides a rough indication of the energy efficiency gains induced by the policy. Energy intensity declines between 8% and 12% in the majority of the models. In contrast to the output comparisons, the changes in energy intensity were remarkably consistent across models and recycling scenarios. The patterns of decline are consistent at the $50 tax level with the magnitude increasing from 8–12% to 16–20%. Total energy efficiency improvements (i.e., including the baseline improvements) range 12–44% and 32% on average in the lump-sum scenario relative to baseline declines ranging 13–39% and 25% on average (see Table 3). Energy efficiency improvements under the carbon policies are significantly greater than the average baseline model behavior, which is comparable with historical energy efficiency improvements of 24% (see Table 3).

### 3.4. Emissions and emissions intensity

**Figure 11.**
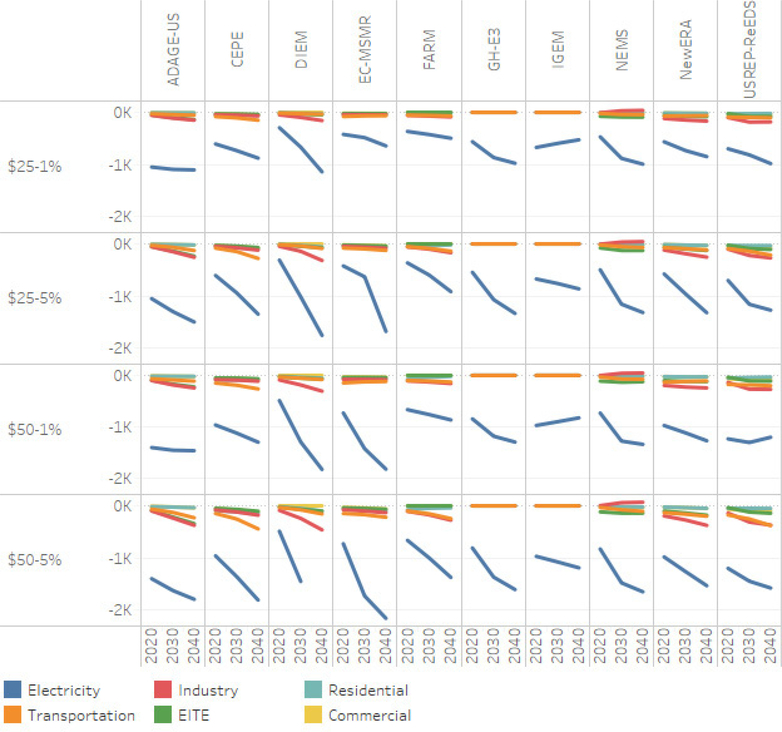
Emissions by sector, level change from reference with lump-sum rebate, 2020–2040 (MtCO_2_/Year).

The carbon tax scenarios have the expected effect of reducing CO_2_ emissions (Fig. 11).^15^ On average, the cross-model comparisons suggest that emissions in 2020 could be 14–23% below the 2020 reference level, while emissions in 2040 could be 21–45% below the 2040 reference level. In the U.S., electricity generation is the largest source of emissions. On average, the cross-model comparisons suggest that the electricity sector contributes 69–72% of total emissions reduction in 2020, while it contributes some 66–77% of total emissions reduction in 2040. Not surprisingly, this sector contributes the most (in absolute tonnes) to emissions reduction. All models and scenarios exhibit this result.

**Figure 12.**
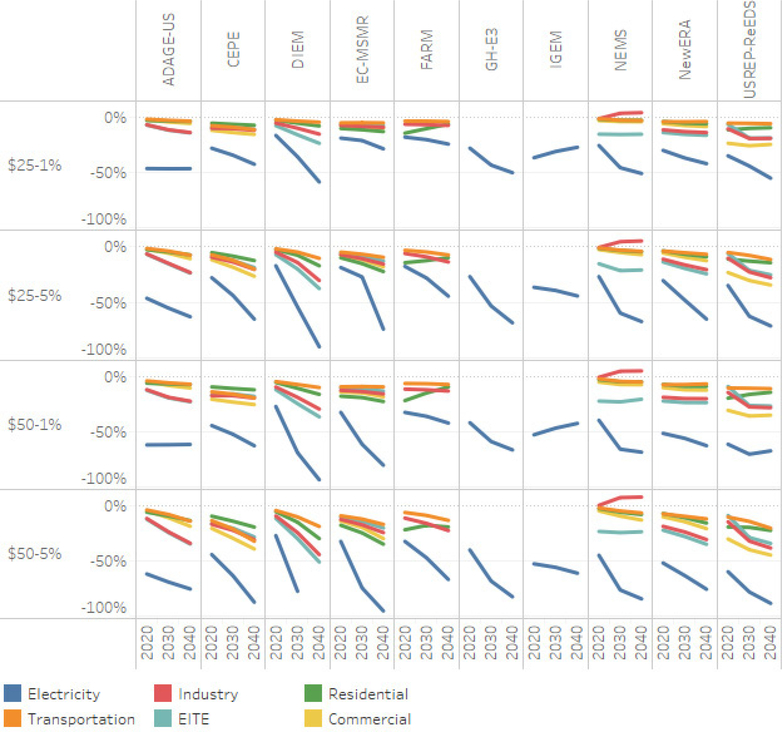
Emissions by sector, percent change from reference with lump-sum rebate, 2020–2040.

In percentage terms, electricity still leads other sectors in emissions reductions. Figure 12 shows for each sector the percent change in emissions relative to the reference case. Across the tax scenarios, each model finds the least cost emissions reductions and the largest total emissions reduction in the electricity sector. Figures 11 and 12 illustrate the importance of the electricity sector in climate policy. (Figures 11 and 12 show results for the lump-sum scenario, other revenue recycling options produce similar emissions trends). Some of the model’s output approaches 100% emissions reductions from the electricity sector in some scenarios.^16^ However, these results risk overshadowing the substantial emission reductions achieved across the transportation, industrial, residential and commercial sectors. We examine these sectoral results in greater detail below.

#### Electricity

**Figure 13.**
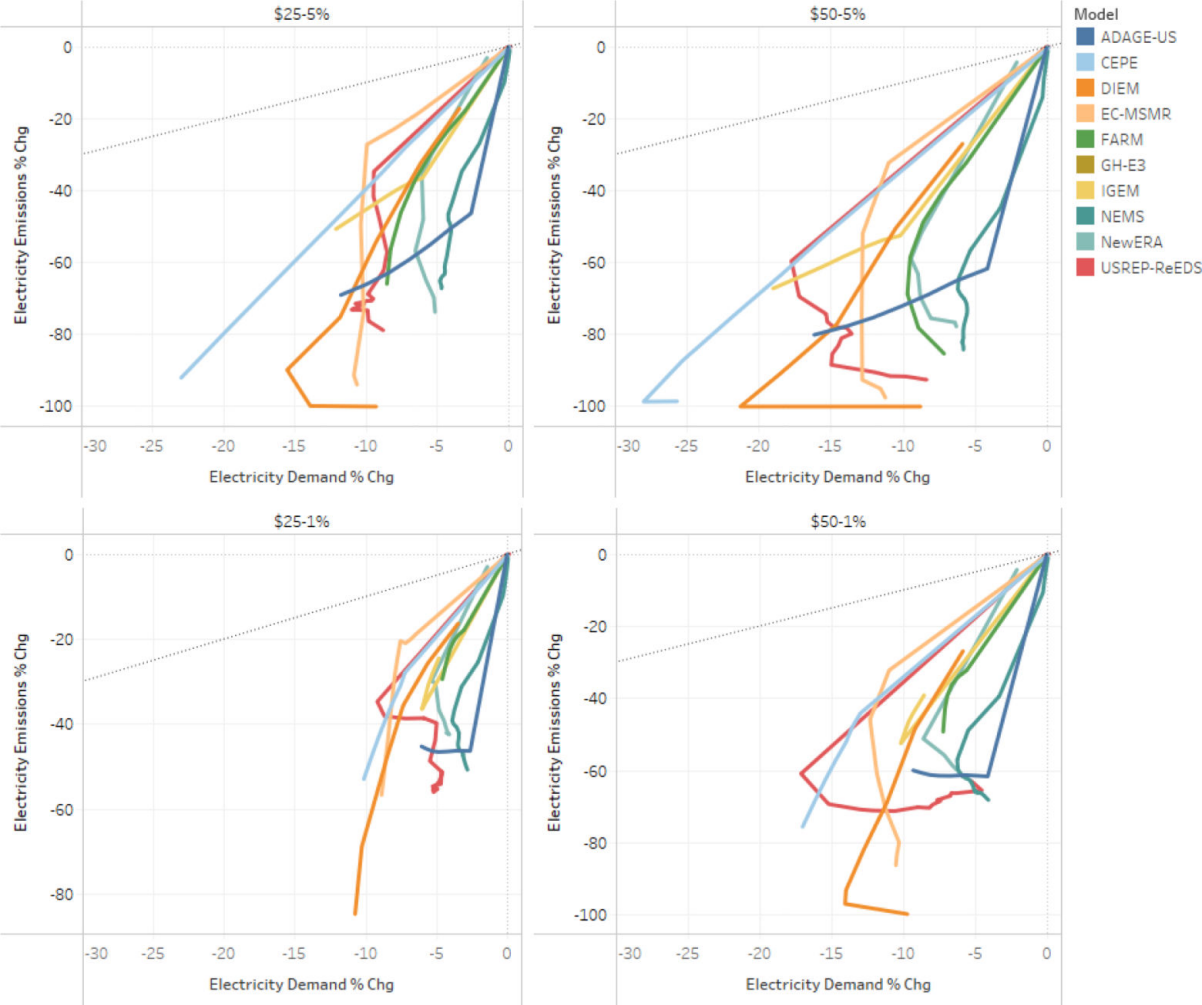
Electricity sector emissions intensity and final energy change.

**Figure 14.**
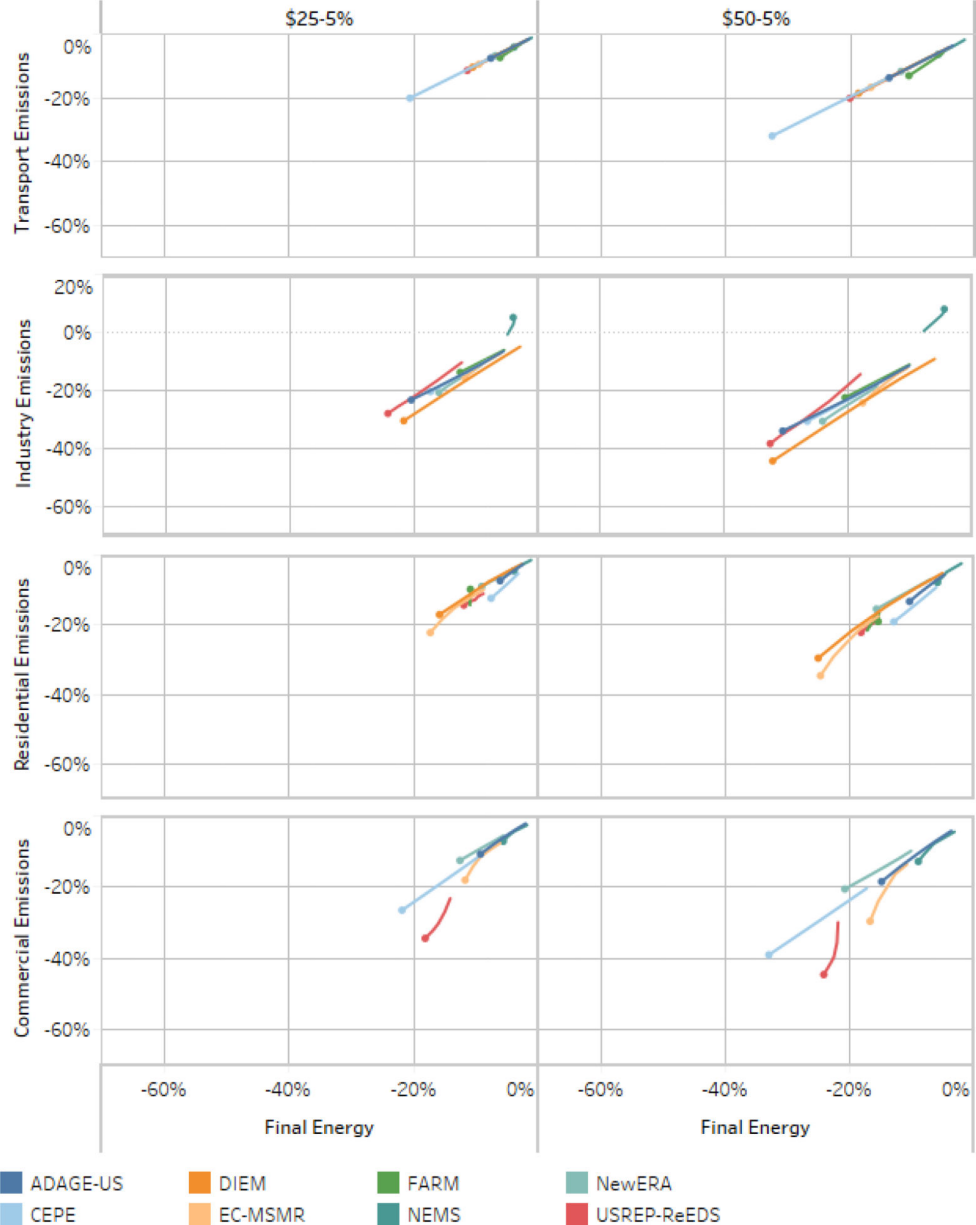
Transportation, industry, residential and commercial sector final energy and emissions % changes, lump-sum rebate scenario, 2020–2040.

The electricity generation sector emitted 1901 Tg CO_2_ in 2015, which was 38% of the US total emissions of CO_2_ (EPA, 2017). The electricity sector emits the most CO_2_ of any sector. Given its reliance on coal, the electricity sector has the greatest potential to decrease its CO_2_ emissions under the carbon tax scenarios. The electricity sector has the lowest-cost mitigation opportunities and therefore is most responsive. The policies were assumed to begin in 2020, but models anticipatory behavior (varies by model) can lead to smoothing emission mitigation investments over time. Figure 13 shows the percentage change from reference in CO2 emissions and final energy used in electricity generation across the models and tax scenarios. Figures 13 and 14 plot the time path of energy and emissions intensity (three dimensions) in a two-dimensional plane. The charts read chronologically from right to left, top to bottom, reflecting the percentage reduction in emissions and energy use. A dashed reference line is included that represents equal percentage reductions, which could be interpreted as a reduction in scale. The modeled electricity sector results fall below this reference line. This indicates that emissions from the electricity sector decline relative to the reference more than final energy use, reflecting decarbonization, for example through fuel switching.

The CEPE and DIEM models fully decarbonize under the $50 at 5% scenario and then the electric sector begins expanding to decarbonize end-use sectors, resulting in an “L”-shaped graph with the slope representing the rate of decarbonization. The FARM, N_ew_ERA, NEMS, and ADAGE-US models seem to follow a steeper decarbonization paths than the remaining models. In 2030, emissions reductions range from 35% in the $25–1% scenario up to 67% in the $50–5% scenario.

#### Non-Electricity Emissions

Transportation is the second largest source of CO_2_ emissions in the U.S., with 1736 Tg CO_2_ in 2015; this represents 35% of total U.S. emissions (EPA, 2017). Compared to the electricity sector, the transportation sector is represented in far less detail in these models (see the first panel of Fig. 14; 2050 is marked with a dot, except for NEMS, for which 2040 is marked as the end of its simulation). In the carbon price scenarios, the models find emissions reductions only through reductions in scale and the percentage declines in emissions track percentage declines in final energy in transportation. These model results do not include low-emissions transportation options or electric cars. In 2030, the cross-model emissions reduction averages are 4% in the $25–1% scenario, up to 11% in the $50–5% scenario. One model, CEPE, shows over a 40% reduction in transportation emissions and fuel use.

The Industrial sector is the third largest source of CO_2_ emissions in the US, emitting 758 Tg CO_2_ in 2015; this represents 15% of total U.S. emissions.^17^ The second panel of Fig. 14 illustrates that the industrial sector has limited ability to decarbonize.

The emissions and final energy paths stay close to the reference line but mostly fall below it, meaning that emissions tend to fall slightly faster (in percentage terms) under the tax scenarios than total final energy use. In 2030, the cross-model emissions reduction averages are 9% in the $25–1% scenario, up to 19% in the $50–5% scenario. One model, NEMS, finds an immediate reduction of final energy use and emissions for the industrial sector, followed by a return to slow growth (Arora et al., 2018).

The residential sector emitted 319 Tg CO_2_ in 2015; this represents 6% of total U.S. emissions (EPA, 2017). The models show a diversity of approaches in modeling emissions from the residential sector, and the cost and availability of emissions reduction strategies, such as energy efficient appliances. Some of these produce “kinks” in the graphs. Generally, the model results for the residential sector in the third panel of Fig. 14 reflect some of the decarbonization in the electricity sector, along with energy efficiency and fuel switching. In 2030, the cross-model emissions reduction averages are 7% in the $25–1% scenario, up to 14% in the $50–5% scenario.

The commercial sector emitted 246 Tg CO_2_ in 2015; this represents 5% of total U.S. emissions (EPA, 2017).^18^ Generally, the model results for the residential sector reflect some of the decarbonization in the electricity sector, along with energy efficiency and fuel switching. In 2030, the cross-model emissions reduction averages are 10% in the $25–1% scenario, up to 20% in the $50–5% scenario.

### 3.5. EITE industries competitiveness and emissions leakage

Imposing a carbon price on emissions would increase the costs of using fossil fuels and hence the cost of producing a good. Higher costs of producing goods would translate to an increase in the price of the good faced by consumers. Energy intensive sectors, which generate a large share of the total industrial emissions and embody much higher emissions per output relative to other sectors of the economy, will face a relatively greater burden of higher energy costs. Moreover, a broad-based policy, such as a carbon price program, applied to all fossil fuels and all sectors of the U.S. economy would raise the production cost of all goods relative to goods produced in other regions of the world that face less stringent carbon constraints. Increases in the costs of producing goods and services are likely to place the U.S. at a comparative disadvantage in international trade relative to nonpolicy regions. This erosion of competitiveness of U.S. energy intensive sectors reduces exports, increases imports, and contracts domestic production, all else being equal. In addition, if emissions regulations are imposed unilaterally, “emissions leakage” would likely occur. Emissions leakage occurs when production in less carbon-constrained regions becomes more price competitive with production facing the expense of a carbon constraint.

Emissions leakage is mitigated to the extent other regions do implement comparable carbon constraints, and many have (see Fig. S1 in the appendix); however, one limitation of the results presented here is that they assume that no other trading partners have regulated carbon emissions in their current policy baseline projection.^19^ In all, international competitiveness of U.S. EITE industries and potential emissions leakage depends upon several factors. Some of the main factors that influence leakage are total emissions, exposure to foreign competition, and emissions intensity (CBO, 2013). Although the two participating models, which have an international dimension, have not explicitly included the NDC targets for rest of the world regions in their baselines, it is recognized that countries are implementing policies that are consistent with the attainment of their NDC targets. In fact, at least one of the models (EC-MSMR) explored the implications of countries acting on their NDC. Recent projections for China and India, in particular, also demonstrate that these two countries’ intensity targets are almost or fully achieved in the baseline as part of their NDC targets. While this paper notes that there will be terms of trade effects as a result of other countries taking on NDC or other carbon targets but the first-order effect on the U.S. will come from its own carbon policies (World Resources Institute, 2016; Liu et al., 2017).

Given the importance of the leakage issue for policy makers in connection to carbon pricing policy, the paper explored how the carbon pricing policies that framed the EMF 32 cross-model comparison, impacted leakage. This paper only reported results from two models, as these are the only models that are of global in nature and results from these two models offer insights on leakage. Despite the fact that only two models reported results, it is instructive to report the leakage implications highlighted by these two models. Given that only two models are of a global nature, it should be noted that these results should be not interpreted as an outcome of MIP but rather as a potential consequence of carbon policy, especially when modeling unilateral carbon policy.

As illustrated in Fig. S13, EITE emissions share is the smallest for the DIEM model (about 20%) while it is the largest for the ADAGE-US model (due in part to different sectoral definitions). Almost 90% of the total industrial emission in the ADAGE-US model is accounted by the EITE industries. N_ew_ERA and USREP-ReEDS models’ EITE emissions share is about 40% while for CEPE, EC-MSMR, and NEMS emissions share ranges from about 60%–70%.

**Figure 15.**
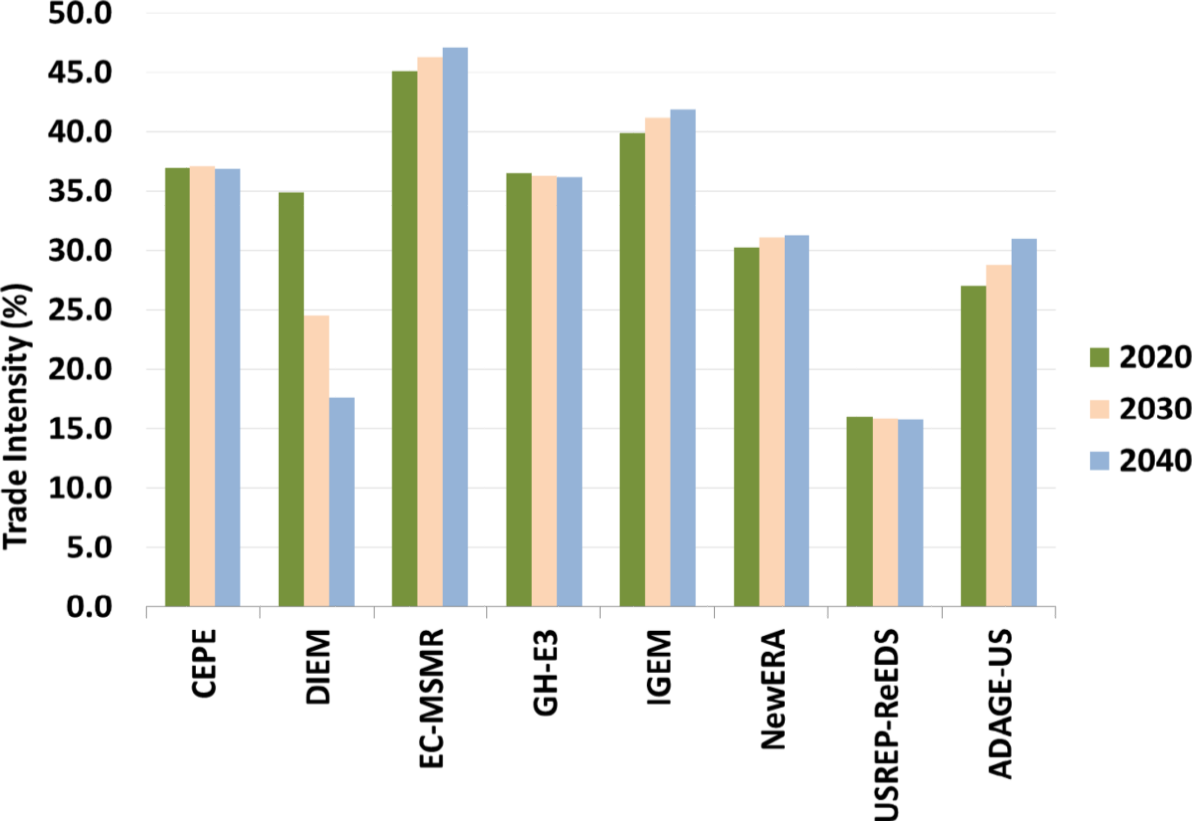
EITE industries trade intensity (%).

Goods that are widely traded are generally impacted by trade spillover effects and subject to international competitiveness when the relative price of goods changes. Trade intensity, defined as the ratio of the sum of exports and imports to the market size, indicates the degree of the industry’s trade-exposure. Figure 15 shows the baseline trade intensity for the EITE industries across different models. Across all models, trade intensity in the baseline for the EITE industries ranges from 15% to 45%. DIEM and USREP-ReEDS models have low trade intensities relative to other models.

The baseline emission intensity of EITE industries spans a wide range across the models. NEMS, N_ew_ERA, and ADAGE-US models have the highest emissions intensity, followed by EC-MSMR, CEPE, and DIEM models. USREP-ReEDS has the lowest intensity. All models exhibit decreasing emissions intensities over time, indicating that the EITE production is rising faster than its emissions. The USREP-ReEDS model emissions intensity is the smallest among all the models at 0.05 MMTCO_2_ per $billions of output (see Fig. S14), since the production base is significantly larger than other models. EITE industry output for the USREP-ReEDS rises from about $6 trillion in 2020 to more than $10 trillion by 2040. In contrast, EITE industries production in the N_ew_ERA model is about $1.5 trillion in 2020 rising to $2.6 trillion by 2040 resulting in relatively higher emissions intensities. EITE emissions intensities for the NEMS and ADAGE-US model are also similar to the N_ew_ERA model (between 0.2 and 0.3 MMTCO_2_ per $billion).

EITE industries, being trade exposed and having relatively high emissions intensity, would be impacted as the cost of production for this sector is higher under a carbon price. The extent to which EITE industries are impacted will depend upon the percentage increase in its costs due to the carbon price.

#### Trade Impacts on EITE Industries

In the absence of carbon restrictions on EITE industries in the rest of the world, the U.S. EITE industries would face a disproportionate increase in the price of EITE industries’ goods compared to EITE prices in other unregulated regions. The modeling results suggest that, with no carbon policy in the rest of the world and the U.S. imposing carbon pricing unilaterally, demand for U.S. EITE goods would decline, driving a reduction in exports of EITE goods. The extent of EITE exports depends upon how easily the U.S. can shift from domestic supply to exports, which in turn depends upon U.S. supply elasticity and rest of the world demand elasticity for U.S. EITE goods. The impacts are much more pronounced as energy costs increase in conjunction with the rise in the carbon price.

**Figure 16.**
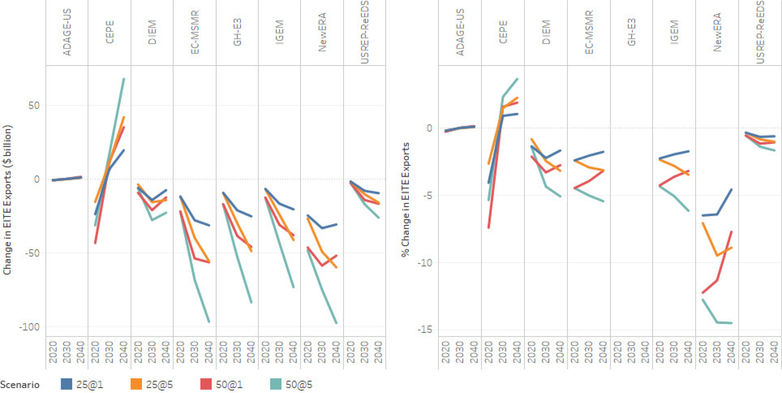
Change and percentage change in EITE industries exports relative to the baseline.

The two panels of Fig. 16 depict level and percentage changes in exports of EITE industries. All models except CEPE and ADAGE-US show reductions in EITE exports relative to the baseline for all carbon price paths. There is minimal change in EITE exports in the ADAGE-US model; while in the CEPE model the change in EITE exports is negative in 2020 and positive thereafter.

Across all the models that show a decrease in the exports of EITE, the reduction ranges from about 1% ($5 billion) for the $25–1% carbon price path in 2020 to about 14% ($50 billion) in 2040 under the $50–5% carbon price path. EC-MSMR and CE-MSMR, E3, IGEM, and N_ew_ERA models show that largest reduction in EITE exports, while DIEM, USREP-ReEDS, and ADAGE-US models show the least amount of EITE exports impacts. The mechanism for the increase in the value of exports of EITE goods in the CEPE model is two-pronged. First, a carbon tax induces a change in the composition of industry output expanding the output of some sectors as production inputs (intermediate inputs and fully mobile capital and labor) are relocated towards sector with comparably low carbon intensities. Second, as the carbon tax reduces income and hence demand for domestic goods, and given the small-open economy assumption, which implies that international demand for US exports is perfectly price-elastic, higher sectoral outputs are re-channeled to foreign markets. Including international competitiveness effects (i.e., relaxing the small-open economy assumption) and sector-specific primary factors of production and capital adjustment costs would likely dampen or reverse the overall effect on exports. In general, the carbon price adder leads to an increase in the producer price of EITE resulting in reducing exports of EITE. Although export demand for EITE decreases, higher EITE export price leads to higher export value of EITE with carbon policy.

**Figure 17.**
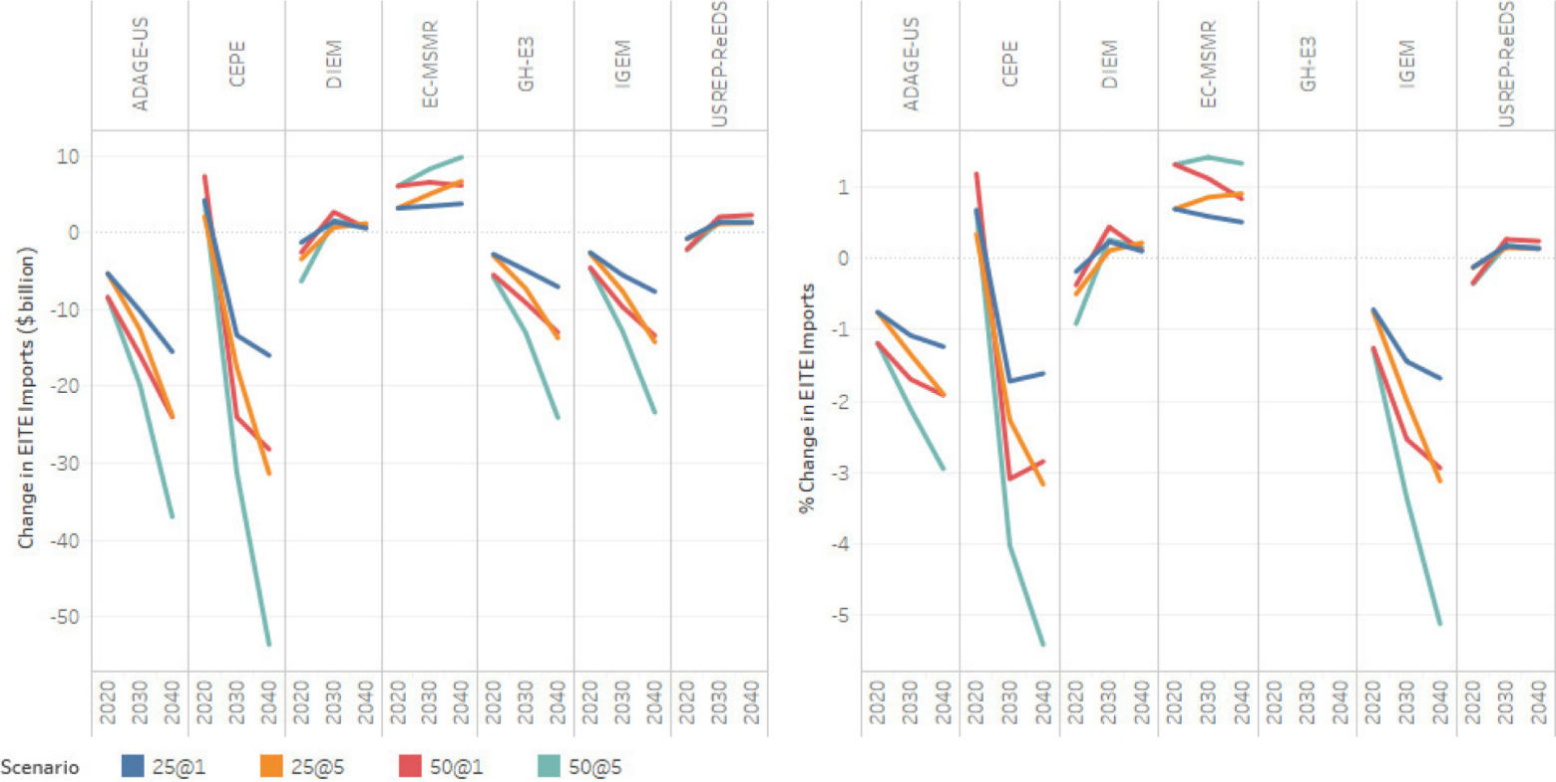
Change and percentage change in EITE industries imports relative to the baseline.

EITE imports on the other hand show mixed results across the models, see Fig. 17. The CEPE, GH-E3, IGEM, and ADAGE-US models depict reduced EITE imports to the U.S. relative to the baseline, while EC-MSMR, N_ew_ERA, and USREP-ReEDS models depict increase in imports of EITE goods relative to the baseline. The change in imports depends upon differences in the relative prices between imported and domestic goods and the ability for imports to displace domestic production. The elasticity of substitution between imported and domestic goods (Armington elasticity) influences the ability for imports to displace domestic goods. For models that show an increase in imports, it suggests that the Armington elasticity is relatively high such that the relative price difference between U.S. and the rest of the world (ROW) causes imports to displace domestic production. This results in an increase in imports. Import of goods and services is also influenced by their total demand in the market. For CEPE, GH-E3, IGEM, and ADAGE-US models decrease in total output is the greatest compare to other models leading to lower labor and capital income to households (see Fig. 7). Lower income to households in these models results in decrease in total demand including demand for import. Hence, these models result in lower imports of EITE goods.

#### Total Emissions and EITE Emissions Leakage Rate

A carbon price affects fossil fuel economics not only within the region the price is implemented but also in the rest of the world through spillover effects from trade. Carbon policy in the U.S. changes the economics of fossil fuels by making it costlier relative to nonfossil energy sources. As already discussed in previous sections, demand for fossil fuels drop leading to a reduction in the producer price of fossil fuels. Price of export goods from the U.S. rises while the rest of the world benefits from lower global energy prices. Overall production of goods and services declines in the U.S. as the expense of higher production of goods and services, in particular EITE goods, relative to the rest of world. This shift in the production of EITE industries from the U.S. to the rest of the world depends upon the incremental demand induced by lower global cost of production and displacement of U.S. production by imports from the rest of the world.

**Figure 18.**
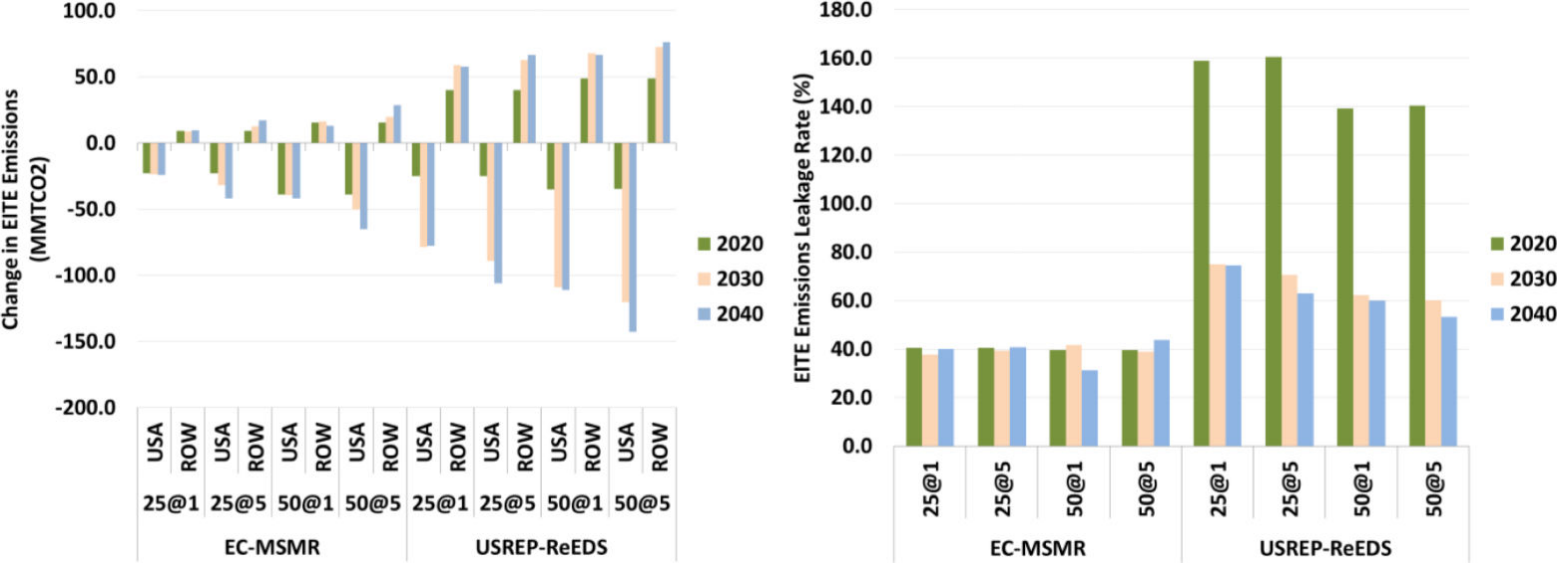
Change in EITE industries emissions in the U.S. and ROW (MMTCO_2_) and leakage rate (%).

Figure 18 shows changes in emissions from the EITE industries and its corresponding leakage rate for two models across the carbon price scenarios with lump-sum recycling cases. In both models, emission declines more as the carbon price increases. For the EC-MSMR model in 2020 under the $25–1% carbon price path, EITE emissions decline by 23 million tonnes of CO2 (MMTCO2); while the rest of the world EITE emission increase by 9 MMTCO_2_. This amounts to a leakage rate of 40%. That is, for every tonne of carbon emissions reduction from the EITE industries in the U.S. there is an increase of 0.4 tonne of EITE carbon emissions in the rest of the world assuming no foreign carbon policies exist. For the EC-MSMR model, the leakage rate increases with the carbon price. For the higher carbon price path, $50–5, in 2040 the leakage rate is about 40% while for the $50–1 the leakage rate is only about 31%. The USREP-ReEDS model leakage rate is significantly higher than for the EC-MSMR model even though EITE emissions intensity is lower for the USREP-ReEDS model. One possible explanation for such a result might lie in the differences in the Armington elasticity assumption and the emissions intensity of the EITE industries in the rest of the world between these two models.

For the USREP-ReEDS model in 2020 under the $25–1% carbon price path, EITE emissions decline by 24 MMTCO_2_ while the rest of the world EITE emissions increase by 40 MMTCO_2_. This amounts to a leakage rate of 160%. That is, for every tonne of carbon emissions reduction from the EITE industries in the U.S. there is an increase of 1.6 tonne of EITE carbon emissions in the rest of the world assuming no foreign carbon policies exist. The rest of world emissions in fact increase more than the decrease in the U.S. in 2020. However, in the 2040 the leakage rate is about 74%. For the USREP-ReEDS model, the leakage rate does not increase with the carbon price. There is less of an increase in rest of the world than decrease in the U.S. on a proportional basis. For this model, the drop in demand in the U.S. induces less imports from rest of the world and hence there is less of an increase in the rest of the world emissions proportionally. In both models, as the carbon price increases the leakage rate declines, although the rate of decline is much smaller in the EC-MSMR than in the USREP-ReEDS model. Moreover, the relative decline over time is much larger for the higher carbon price trajectory ($50–1%) than for a lower carbon price trajectory ($25–1%).

As demonstrated in the GDP section above, higher carbon prices lead to higher reductions in economic activity. This leads to lower demand for goods and services including imports. Lower imports imply less production of goods and services outside the U.S. and therefore lower emissions in rest of the world. The lower leakage rate reflected overtime is influenced by the stringency of the carbon price on demand for imports, especially for energy-intensive goods.

**Figure 19.**
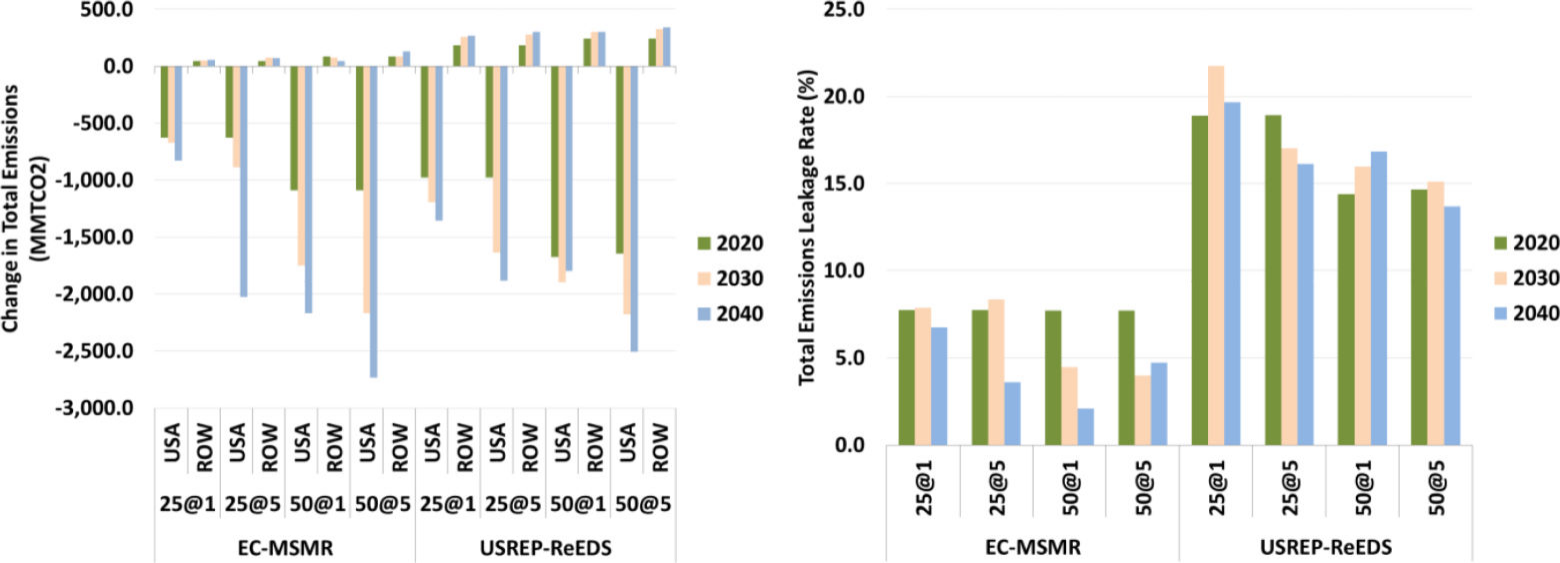
Change in total emissions in the U.S. and ROW (MMTCO_2_) and leakage rate (%).

Figure 19 shows changes in total emissions in the U.S. and the rest of the world and its corresponding leakage rates for two models across the carbon price scenarios with lump-sum recycling cases. In both models, higher carbon prices lead to higher reduction in the U.S. emissions. For the EC-MSMR and USREP-ReEDS models in 2020 under the $25–1% carbon price path, total emissions decline by 630 and 970 MMTCO_2_, respectively; while for emissions in the rest of the world increase by 48 and 185 MMTCO_2_, respectively. This amounts to leakage rate of 8% and 19%. The leakage rates associated with total emissions are much smaller than the corresponding EITE industries leakage rates because the emissions intensity of the rest of the economy is relatively smaller than the EITE emissions intensity, which induces much smaller proportional changes. As with the EITE industries, the total emissions leakage rate for the USREP-ReEDS is double that of the EC-MSMR model.

The leakage rates estimated by the two models, EC-MSMR and USREP-ReEDS, fall within the range projected in Böhringer *et al.* (2012). The study involved 12 modeling teams that simulated a reduction policy of 20% from historical 2004 emission levels of the abatement coalition (developing countries). Across all models, this study projected a leakage rate to range between 5% and 19% with a mean value of 12%. Unilateral emission reduction policies, such as the ones analyzed in this study undermine the efficacy of the goal of the emissions reduction policy by inducing relocation of emissions from U.S. to the rest of the world (leakage) and disproportionately harm energy-intensive and trade exposed industries in the region that take on the policy.

Emissions leakage will be mitigated to the extent that other regions implement similarly restrictive carbon policies. Leakage to unregulated regions can be mitigated by implementing BCAs complementary to carbon policies (Markusen, 1975; Parker and Blodgett, 2008; Arnold, 2013). Under the BCA measure, embodied carbon is taxed from unregulated regions while the price paid for embodied carbon in exports from regulated regions is rebated back. This provides a level playing field by limiting the relative price differential between the regulated and unregulated regions. BCAs mitigate emissions leakage and harm to EITE industries in regulated regions. Böhringer *et al.* (2012) results indicated that BCA as a complementary policy is effective in reducing the mean value of EITE leakage rate from 12% to 8%.

#### Impacts of Revenue Recycling on EITE Trade

**Figure 20.**
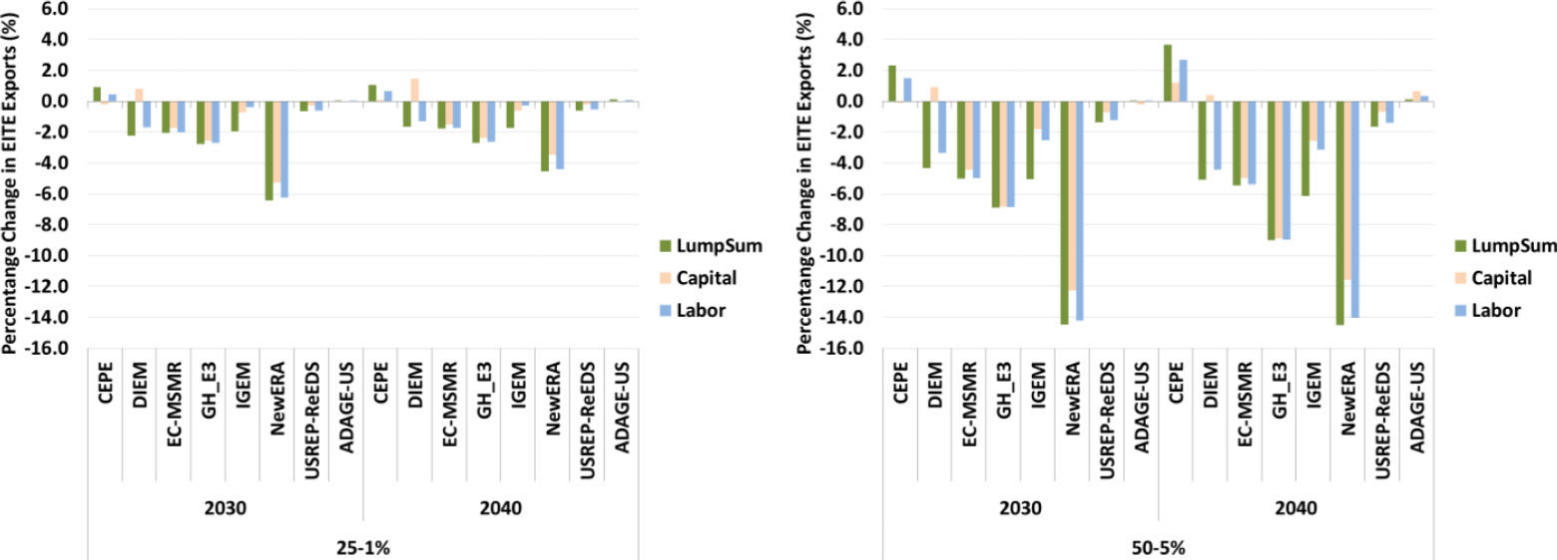
Percentage change in EITE exports (%) for $25–1% and $50–5 revenue recycling scenarios (lump-sum, capital, labor).

Figure 20 illustrates impacts on EITE exports of carbon revenue recycling through lump-sum, capital income and labor income for the $25–1% and $50–5% scenarios. Revenue recycling does have an impact on industry trade and competitiveness. The mechanisms by which reducing capital and labor income tax rates can improve the economy are clear. Reducing distortionary tax rates on factors of production benefits the economy as a whole in several ways. Reducing these tax burdens results in more income for consumers and businesses and increases their incentives to work and invest, all of which would have positive economic consequences. Lower tax rates also mean that the cost of producing goods and services are lower, thereby improving cost competitiveness of domestic industries. Moreover, tax interaction effects between carbon and factor taxes are also reduced. The main drawback to tax rebates is in the distribution of policy impacts. For capital tax recycling in particular, lower-income households tend to be disproportionately impacted by the policy (see Caron et al., 2018).

All model results indicate that reducing factor tax rates through recycling reduces some of the losses in EITE exports compared to the lump-sum recycling case. With recycling, lower capital or labor tax rates improve the cost competitiveness of EITE industries, resulting in a smaller impact than under the lump-sum case, which does not provide incentive to invest. DIEM, IGEM, and N_ew_ERA models show the largest reduction in loss in EITE exports. Results for some models (CEPE, DIEM, and ADAGE-US) show that capital tax recycling leads to positive changes in EITE exports compared to the lump-sum case. These results indicate that the negative effect of a carbon tax is outweighed by the reduction in capital taxes, improving EITE cost competitiveness, and increasing exports compared to the baseline. Although reductions in labor tax rates improve the trade position of the EITE industries, the impacts are much smaller than for capital tax recycling. It should be noted that the effect of recycling depends upon the underlying tax rates, revenue available for recycling, and tax interaction effects.

**Figure 21.**
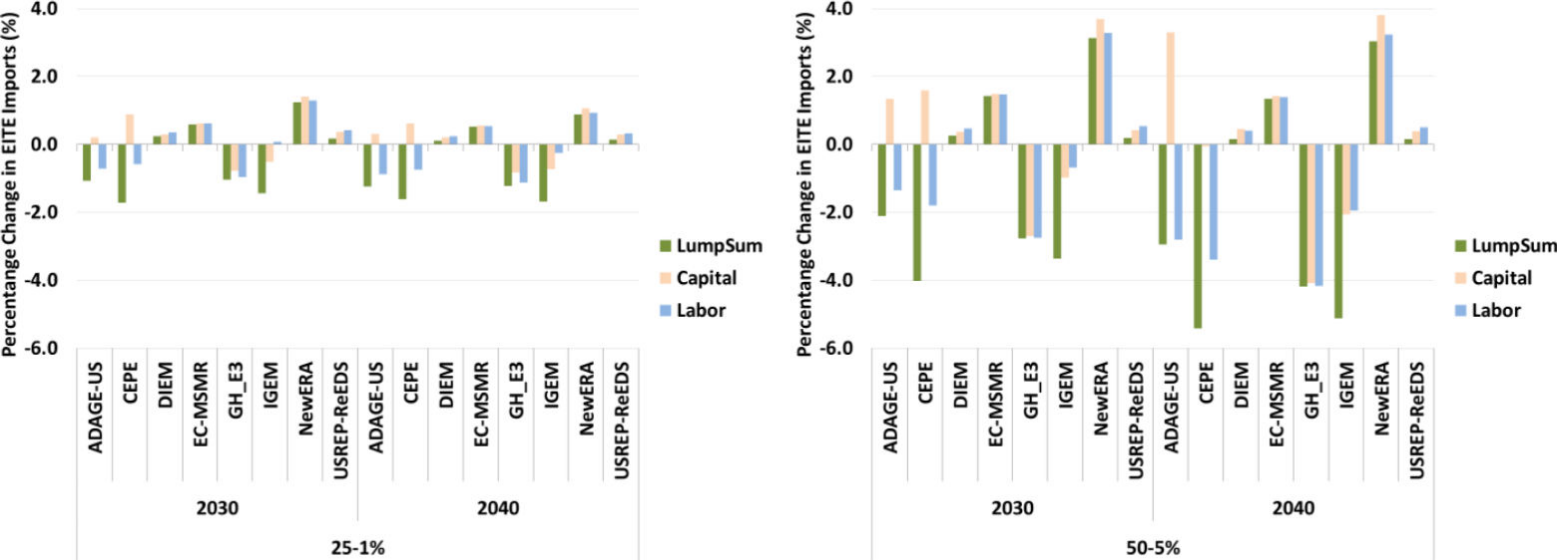
Percentage change in EITE imports (%) for $25–1% and $50–5 revenue recycling scenarios (lump-sum, capital, labor).

Finally, Fig. 21 illustrates impacts on EITE imports of carbon revenue recycling through lump-sum, capital taxes, and labor taxes for $25–1% and $50–5 scenarios. As with the EITE exports, EITE imports are also impacted by the revenue recycling mechanism. Reducing tax burdens results in more income for consumers and businesses and higher imports relative to the lump-sum case.

## 4. Discussion

### 4.1. Key takeaways

Options available to policymakers differ in their impacts on various sectors of the economy. Multiple policy options must be vetted to better comprehend their economic effects, both negative and positive. Policymakers’ preferences will of course vary in the costs they can accept and the benefits they value. There will be no “silver bullet” policy that suits all policy makers. This paper has highlighted some key nuances in how carbon policy options may impact sectoral output, energy use, and emissions. These results can provide valuable guidance for all policy makers in their assessments.

The EMF32 cross-model comparison results illustrate that without a new carbon tax, primary energy demand rises steadily along with economic expansion throughout the forecast period. However, if a carbon tax is implemented, the demand for emissions-intensive energy sources (like coal and oil) give way to less emissions-intensive sources (like renewables and natural gas). All eleven models show how primary energy use within each economic sector adjusts in response to a carbon price. Here the electricity sector has the most flexibility to fuel-switch away from emissions-intensive fuels, and given that it has the lowest abatement cost, it takes on a dominant share of total abatement (compared to the residential, industrial, commercial, or transportation sectors).

Sectors of the economy vary in their ability to affect emissions reductions through fuel switching. Electricity is the largest source of emissions and the models show more fuel-switching in this sector compared to other economic sectors. The second-largest source of emissions, transportation, exhibited little to no reduction in emissions intensity, although this sector is not represented well enough in most models to capture such behavior (e.g., electrification). As an indicator of fuel switching, Table 4 presents the percent reductions in the aggregate emissions factor of total final energy. The variation across recycling schemes is slight, with the largest reduction in intensity occurring under capital tax recycling.

Table 4 also shows changes in energy intensity of output, which is comparable but smaller than the emissions intensity for energy, and largely insensitive to the different recycling mechanisms. Reporting emissions intensity with respect to output (as opposed to GDP, as is common) enables comparison with Section 3.3. Of the three emissions components, total output shows the smallest average percent change relative to the baseline, but that percent change is relatively more sensitive to the recycling mechanism, with much smaller total output declines in the capital tax recycling scenario. A similar pattern was evident across models for EITE output (see Fig. 9).

Emissions reductions are also affected by changes in output. In all models, total output reacts most negatively to a carbon price on emissions-intensive fuels when carbon revenue is recycled lump-sum. Model results highlighted how output declines were generally larger for EITE sectors than for total output. Percent declines were smaller for GDP than for total output. While output results are an important aspect of understanding emissions behavior, GDP is a better measure for conveying the economic impact of the policy on households and individuals. In percentage terms, output changes contribute the least to changes in total emissions by two orders of magnitude relative to emissions factors and energy intensity (see Table 4).

Most model results show a reduction in exports from EITE industries if a carbon price is implemented, unilaterally in these scenarios. Results for imports are mixed. Each model’s elasticity of substitution between imported and domestic goods plays a key role here. Unilateral carbon policy would be particularly subject to emissions leakage. That is, a carbon price implemented unilaterally in one region will support a shift in production away from that region to a region with no carbon price. Critics of a unilateral carbon price cite emissions leakage as “exporting” pollution. This ultimately undermines the original “raison d’être” (or goal) of an emissions reduction policy. As modeled, the policies considered here did not account for carbon policies in place currently or during the modeled period elsewhere in the world, which would mitigate emissions leakage. The considered policies also ignore BCAs, which would further mitigate emissions leakage.

### 4.2. Limitations and future research

The conclusions drawn here have identified certain limitations that further research could help address. To assess policy leakage more robustly, models would ideally have global representation of multiple different trading regions and countries, their carbon policies, and potential border-carbon adjustments. While our focus was on EITE sectors as a whole, further research could help identify those individual sectors that might be most vulnerable to adverse impacts from carbon policies. Models with more detailed representations of countries, their carbon policies, and energy-intensive sectors, could reveal important nuances with regard to how terms of trade effects might influence policy leakage.

Models in this study were not able to capture all abatement options available to the U.S. economy. In particular, electrification options in the transportation and industrial sectors were largely absent as this remains an emerging phenomenon with significant uncertainty. With electric vehicle technologies becoming more cost competitive in recent years (IEA, 2017), continued cost improvements leading to further electrification could significantly alter the emissions intensity of transportation, the U.S.’ second largest source of emissions, and indirectly alter the emissions intensity of electricity, the U.S.’ largest source. Improved characterization of how vehicle, charging infrastructure, and electricity costs will influence electric vehicle adoption could significantly alter modeled abatement under carbon policies.

The economic and environmental measures we focused on in this paper are a subset of those necessary to determine whether carbon policies should be undertaken. Measures such as GDP or household welfare can speak more directly to the policy costs borne by individual households (see Caron et al., 2018). General equilibrium welfare measures may still obscure significant economic impacts stemming from sectoral unemployment and labor market transitions, as general equilibrium representations of these economic phenomena are an active area of research.

We have not directly addressed the economic benefits of carbon policies in our analysis. While emissions abatement provides a relative indication of which policies are likely to generate more benefits, physical units of abatement offer no guidance on the level or marginal economic value of that abatement such that one could perform cost-benefit assessments. Moreover, research indicates that co-benefits from climate policy may be substantial (e.g., West et al., 2013; Woollacott, 2018), but this remains a valuable area of further research. This is particularly true given the spatial and potential economic heterogeneity of co-benefits, which could significantly influence a policy’s net benefits.

## 5. Conclusion

This multi-model study revealed that carbon tax policies achieve the primary goal of reducing GHG emissions through a combination of substitution, efficiency improvements, and output reductions. Although different recycling mechanism had material impacts on economic outcomes, emissions reductions were generally insensitive to them.

Substitution in the form of fuel switching from carbon-intensive fuels such as coal toward less carbon-intensive energy sources such as gas and renewables is apparent across the models analyzed here with the exception of models with cost-competitive CCS, which dramatically reduces coal’s carbon intensity. Models also achieved emissions reductions through substitution away from domestic, energy-intensive sectors. The resulting change in the sectoral composition of the economy supported aggregate energy efficiency improvements. Aggregate efficiency improvements contributed slightly less to emissions reductions than fuel substitution. Least of all, output reductions contributed two orders of magnitude less to emissions reductions than fuel switching or energy efficiency improvements.

Revenue recycling influences aggregate economic measures such as output and GDP. For example, production levels were generally higher under capital tax recycling than other recycling options and household energy consumption varied with recycling mechanism. Emission levels, however, are not significantly influenced by how the carbon revenue is used. Higher taxes and growth rates beget larger emissions reductions. This is helpful guidance to policy makers, who might prefer to consider the level and allocation of carbon revenue separately. Given a tax trajectory to meet emissions abatement goals, policy makers can focus on recycling mechanism to address the economic consequences for certain sectors (e.g., EITE) and households (see Caron et al., 2018).

## Supplementary Material

Supplementary Material

## Figures and Tables

**Table 1.** Sectoral and technological details of the models.

Model name	Covered sectors	Electric sector technology detail	Other important attributes
CEPE (ETH)	5 energy (crude oil, refined oil, gas, coal, elec) 3 demand, 5 nonenergy production	3 fossil, hydro, nuclear, wind	US only
DIEM (Duke)	6 energy (crude oil, refined oil, ethanol, gas, coal, elec), 5 nonenergy, 3 demand, 6 nonenergy production	CGE: 6 conventional (fossil, nuclear, hydro, biomass, wind, solar), 2 CCS (coal, gas)	
EC-MSMR (Env. CC Canada)	5 energy (crude oil, refined oil, gas, coal, electricity) 3 demand, 15 nonenergy production	3 fossil (2 with & 3 without CCS), nuclear, hydro, wind, solar, biomass (w & w/o CCS), geothermal	8 crude oil and oil sands technologies for Canada, bitumen refinery technology for USA
FARM (USDA)	38 production sectors (5 energy, 33 nonenergy)	3 fossil (oil, gas, coal), nuclear, hydro, wind, solar PV, 2 bio-electricity (switchgrass, forest residue), MSW	CCS can be switched on or off for fossil and bio-electricity; land use by 18 agro-ecological zones and 9 crop types
G-Cubed (ANU, Syracuse, Brookings)	20 sectors; 14 energy, 6 nonenergy	3 fossil (coal, gas, oil), nuclear, wind, solar, hydro, other.	
GH-E3 (RFF)	35 sectors, 9 energy, 26 nonenergy	3 generator types: coal, other fossil (primarily gas) and nonfossil wholesale generators, with t/d sector to sell retail	
IGEM-N (Northeastern, DJA)	NAICS-based: 6 energy (coal, oil mining, gas mining, refined petroleum, electric and gas utilities), 30 nonenergy	Fossil fuel energy inputs plus capital, labor and nonenergy materials	US only
NEMS (EIA)	4 supply (coal, oil, gas, renewables); Electricity, Refining; 4 demand-Res., Comm., Ind., Trans.	Plant level detail for existing plants; New plants — PV, CSP, Wind, Coal, Coal w/CCS, NG CT, NGCC, NGCC w/CCS, Nuclear, Hydro, GT, MSW, Biomass; Retrofits — Coal to NG, coal w/CCS	CCS is 90% capture; endogenous capacity retirements
N_ew_ERA (NERA)	12 sectors: 6 energy (oil, gas, coal, refoil, elec, biofuels); 7 nonenergy sectors (ag., manuf., motor veh. manuf., energy int. sectors, services, trucking, other comm. trans.)	7 fossil, 2 CCS (coal, gas), nuclear, 2 bio (landfill, bio-only), 6 renewable (hydro, geo, 2 wind, 3 solar), 2 storage (pump hydro, battery)	Linked model: Top-down macro model fully linked with bottom-up electric sector model.
USREP-ReEDS (NREL, MIT)	5 energy (crude oil, refined oil, gas, coal, elec) 3 demand, 6 nonenergy production	ReEDS: 7 fossil, 2 CCS (coal, gas), nuclear, 2 bio (landfill, bio-only), 5 renewable (hydro, geo, multiple wind & solar classes, technologies), 3 storage (pumped hydro, CAES, battery)	Linked model: Top-down macro model fully linked with bottom-up electric sector model.
ADAGE-US (RTI)	10 sectors, 5 nonenergy and 5 energy.	Fuel-specific	US only; Not running linked electricity model (EMA)

**Table 2.** U.S. carbon tax policy scenarios.

Carbon price path	Revenue recycling option			Carbon price by year				
	HH	K	L	2020	2025	2030	2035	2040
Reference								
$25–1%				$25	$26	$28	$29	$31
$50–1%				$50	$53	$55	$58	$61
$25–5%				$25	$32	$41	$52	$66
$50–5%				$50	$64	$81	$104	$133

**Table 3.** Economy-wide energy intensity improvements, 2020 versus 2040 and historical.

	Average	CEPE	DIEM	EC-MSMR	NEMS	N_ew_ERA	ReEDS-USREP	ADAGE-US
Baseline	−25.2%	−24.8%	−25.4%	−39.3%	−12.6%	−23.8%	−35.5%	−15.0%
Lump-sum	−32.3%	−31.7%	−39.3%	−42.9%	−11.6%	−29.3%	−44.0%	−27.7%
1995 versus 2015	−23.8%							

*Notes*: Historical data are calculated based on the EIA Monthly Energy Review, the Federal Reserve industrial production index, and the average total output reported by the models for the year 2015.

**Table 4.** Decomposition of emissions changes for a $25/tonne tax at 5% growth by recycling scenario.

	Average	CEPE	DIEM	EC-MSMR	N_ew_ERA	USREP-ReEDS	ADAGE-US
Baseline (2020–2040 NPV)							
Emissions/energy (Bn. Tn./EJ)	0.28	0.34	0.21	0.35	0.20	0.30	0.28
Energy/output (EJ/Tril. $)	0.56	0.37	0.84	0.35	0.76	0.45	0.57
Output (Tril. $)	96.1	107.1	79.3	107.5	92.2	98.6	92.0
Emissions (Bn. Tonnes)	13.9	13.7	14.2	13.2	13.8	13.6	14.8
Percent change from baseline							
*Lump-sum*							
Emissions/energy	−12.5%	−13.9%	−12.7%	−11.9%	−10.4%	−10.9%	−15.3%
Energy/output	−10.8%	−9.9%	−8.7%	−7.7%	−11.3%	−17.2%	−10.2%
Output	−0.9%	−1.5%	−0.7%	−0.5%	−0.8%	−0.6%	−1.5%
Emissions	−22.7%	−23.5%	−20.8%	−19.1%	−21.1%	−26.7%	−25.1%
*Capital*							
Emissions/energy	−13.0%	−13.8%	−14.3%	−12.1%	−10.7%	−10.9%	−16.2%
Energy/output	−10.7%	−10.1%	−8.4%	−7.6%	−11.1%	−17.3%	−9.8%
Output	−0.1%	−0.3%	0.6%	−0.3%	−0.4%	−0.3%	0.2%
Emissions	−22.4%	−22.7%	−21.1%	−19.0%	−20.9%	−26.6%	−24.3%
*Labor*							
Emissions/energy	−12.5%	−13.6%	−12.7%	−11.9%	−10.4%	−10.8%	−15.5%
Energy/output	−10.8%	−10.1%	−8.7%	−7.7%	−11.3%	−17.3%	−10.0%
Output	−0.7%	−1.0%	−0.4%	−0.4%	−0.7%	−0.4%	−1.2%
Emissions	−22.5%	−23.1%	−20.6%	−19.1%	−21.1%	−26.5%	−24.8%

## Footnotes

1 https://www.clcouncil.org/wp-content/uploads/2017/02/TheConservativeCaseforCarbonDividends.pdf.

2 http://www.ourenergypolicy.org/wp-content/uploads/2017/07/RFF-IB-17-09.pdf.

3 World Bank and Ecofys (2017).

4 In H.R. 2454, an industry’s energy intensity is defined as its energy expenditures as a share of the value of its domestic production. An industry’s GHG intensity is defined as its total greenhouse gas emissions (including indirect emissions from electricity consumption) times $20 per tonne of emissions, divided by the value of the industry’s domestic production. An industry’s trade intensity is defined as the combined value of its exports and imports as a share of the value of its domestic production and imports.

5 These modeling results reflect impacts from a least-cost solution of meeting a carbon constraint and are not a benefit-cost analysis.

6 The modeling results are presented for the following EMF 32 participating models: CEPE, DIEM, EC-MSMR, FARM 3.1, G-Cubed, IGEM, NEMS, N_ew_ERA, ADAGE-US, USREP-ReEDS.

7 These estimates are for total primary energy averaged across the participating model.

8 See Figs. S2 and S3 for more details.

9 http://unfccc.int/files/national_reports/biennial_reports_and_iar/submitted_biennial_reports/application/pdf/2016_second_biennial_report_of_the_united_states_.pdf (see p. 62).

10 https://www.epa.gov/energy/greenhouse-gases-equivalencies-calculator-calculations-and-references; and https://www.eia.gov/environment/emissions/co2_vol_mass.php.

11 The primary energy by fuel levels for the revenue recycling sensitivity (where half of revenues are used to reduce Capital Income Tax Rate and half are returned in Lump-Sum Rebates to All Households) is not depicted as the primary energy levels are comparable to the other sensitivities. NEMS did not report results for the labor income tax rebate scenario.

12 See Figs. S4–S6.

13 While there were 10 participating models, only seven models had results for the cases discussed in this section.

14 EITE sectors include iron and steel, nonmetallic minerals, chemicals and plastics, and nonferrous metals.

15 All 10 models report electricity emissions. In addition, eight models report emissions for Industry, Residential, Commercial and EITE sectors.

16 Electricity mitigation potential varies by model, depending on prices and technical assumptions about cleaner fuel sources.

17 Industry is also a significant source of non-CO_2_ GHGs, which are not addressed here.

18 Commercial enterprises are also a large source of non-CO_2_ GHGs which are not considered here.

19 Two of the participating models (i.e., EC-MSMR and USREP-ReEDS) reported results for the rest-of-the-world at the country-specific level. EC-MSMR assumes that there is no policy for the other countries. In baselines, the major developed countries (e.g., Canada, EU-28, Japan, Australia, New Zealand, Russia, and other Annex 1 countries) following the GDP and emissions paths reported in their 2015 Biennial Report submitted to the United National Framework Convention on Climate Change, while the remaining countries (such as China, India, South Korea, Brazil, Mexico, and emerging nations) follow their GDP and emissions paths projected in the U.S. Energy Administration’s International Energy Outlook 2016.
