# Supplementary material for: THE IMPACT OF CARBON TAXATION AND REVENUE RECYCLING ON U.S. INDUSTRIES
Source: Clim Chang Econ (Singap). Author manuscript; Available in PMC 2020 Mar 2. (PMC7050298; doi:10.1142/S2010007818400055)
Supplement: Supplementary Material [file NIHMS966723-supplement-Supplementary_Material.pdf]

Supplementary Material

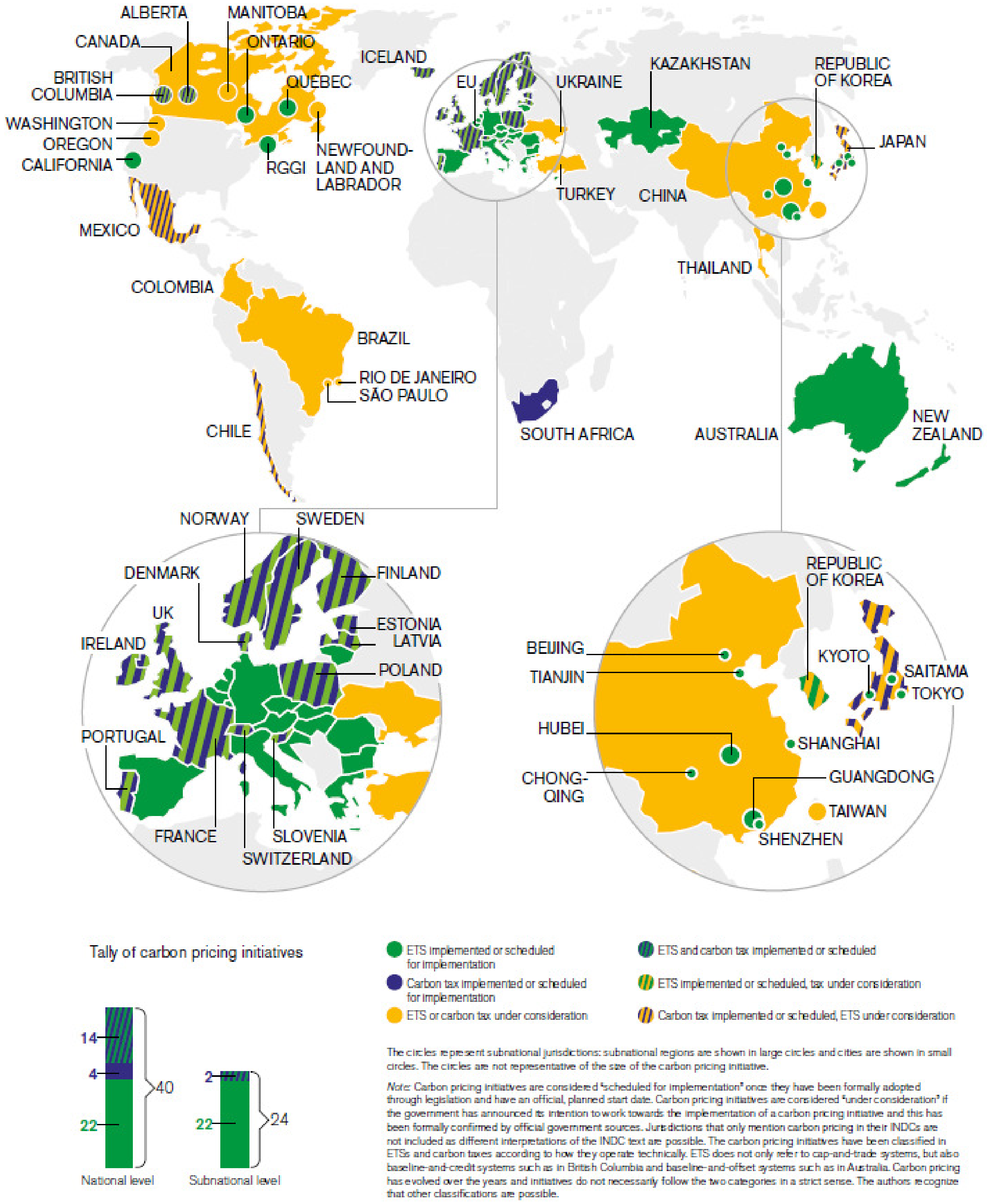

Figure S1: Map of existing, emerging and potential regional, national and subnational carbon pricing initiatives (ETS and tax).

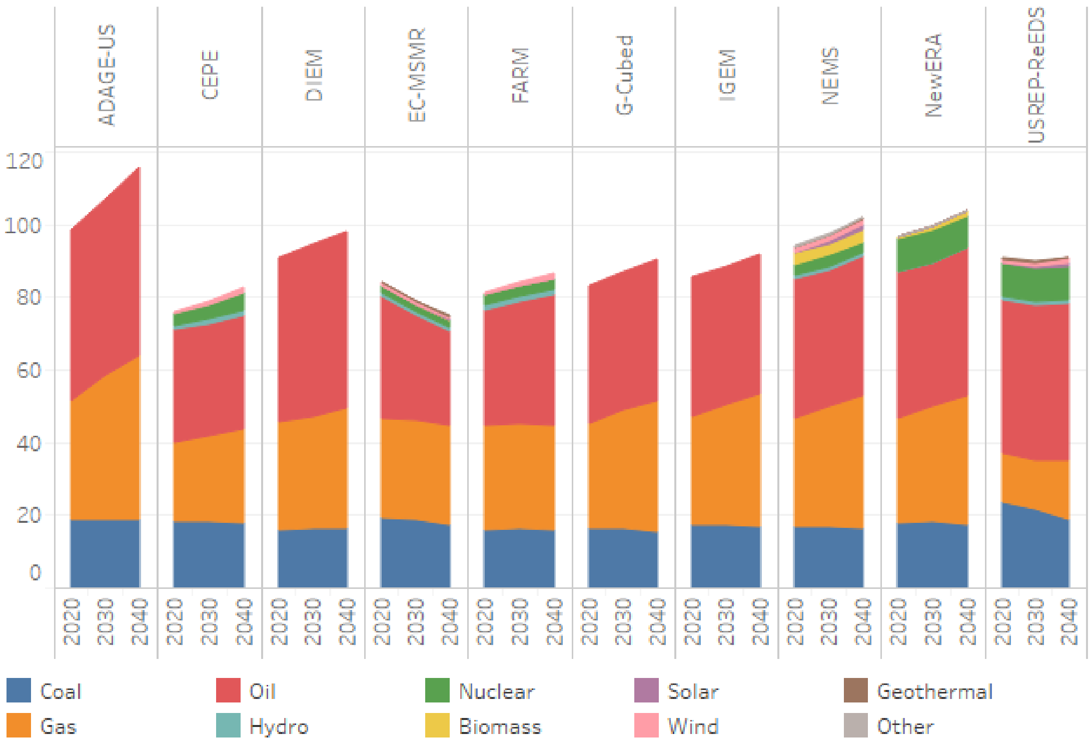

Figure S2: Reference Scenario Primary Energy Demand (Exajoules per Year).

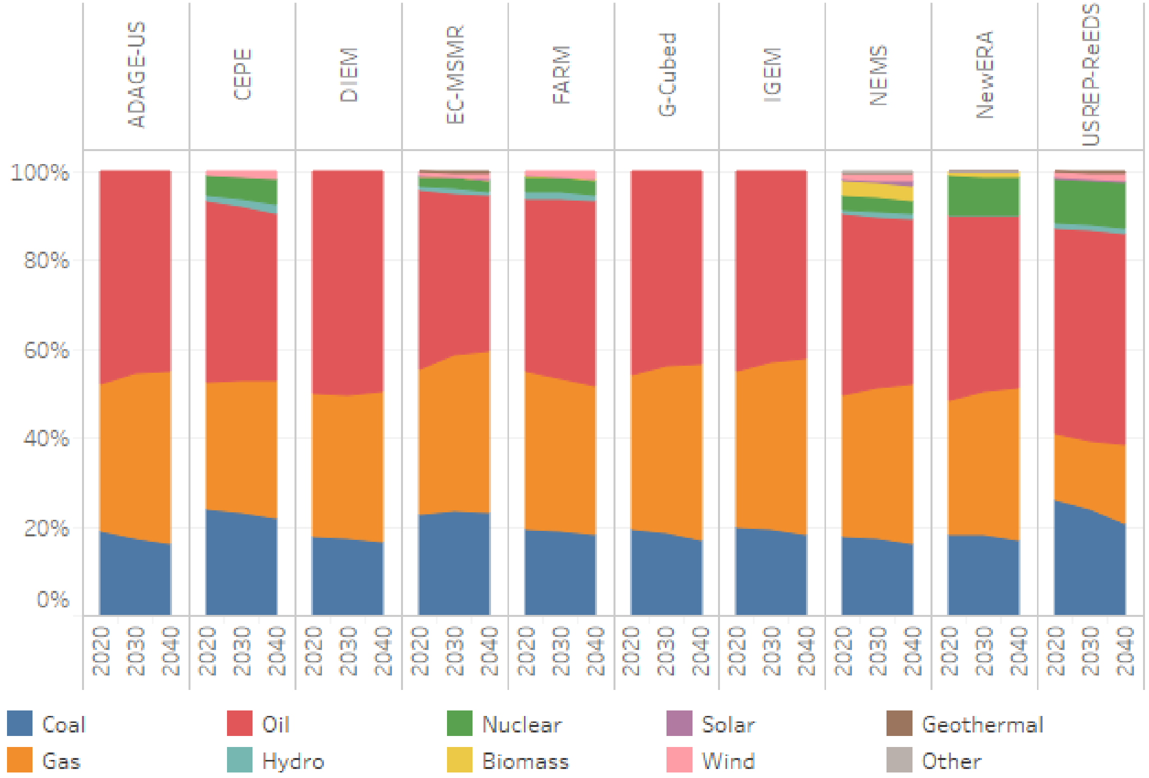

Figure S3: Reference Scenario: Share of Fuels as Total of Primary Energy Usage

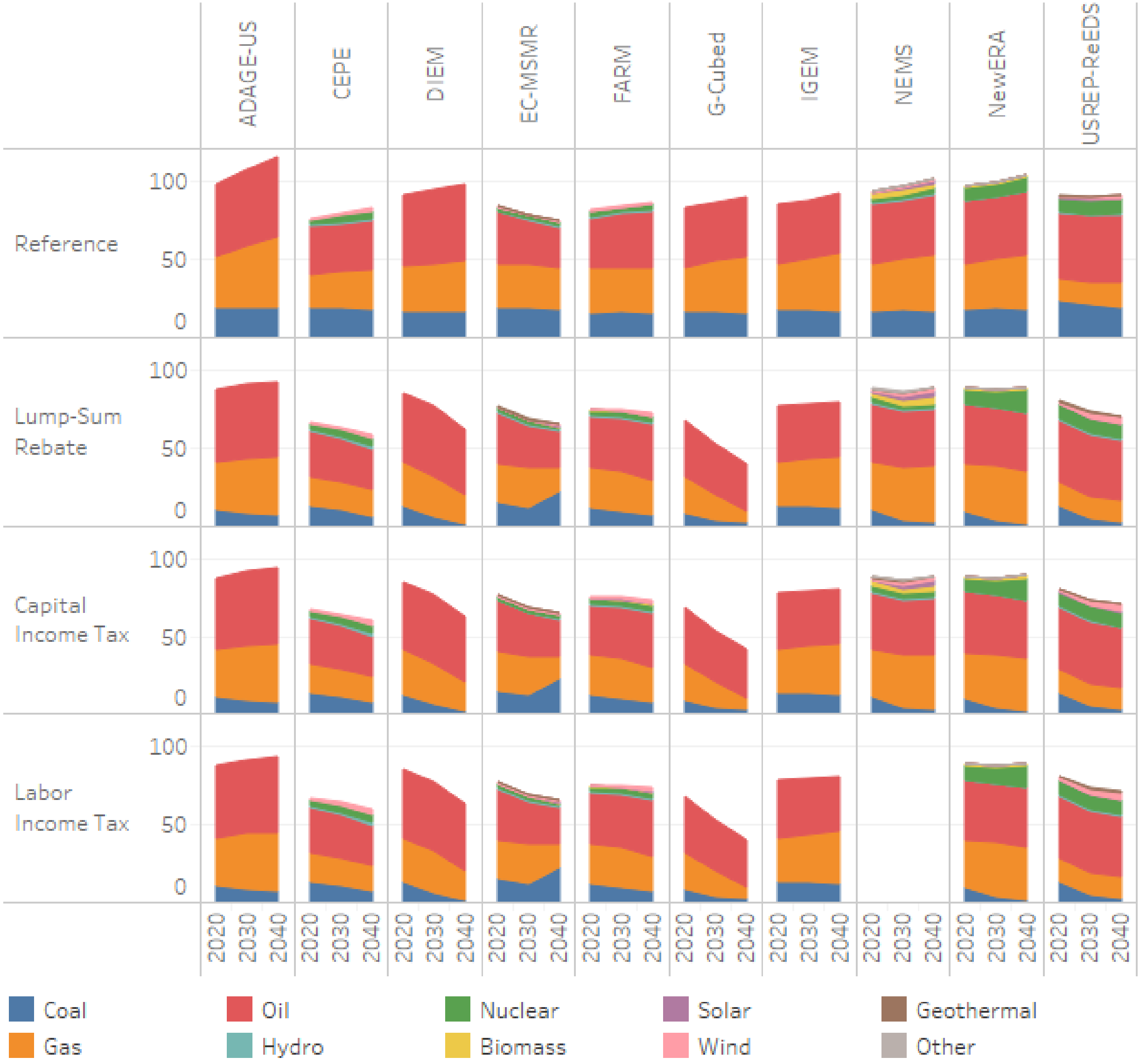

Figure S4: Primary Energy: Impact of Carbon Revenue Recycling (Exajoules per Year)

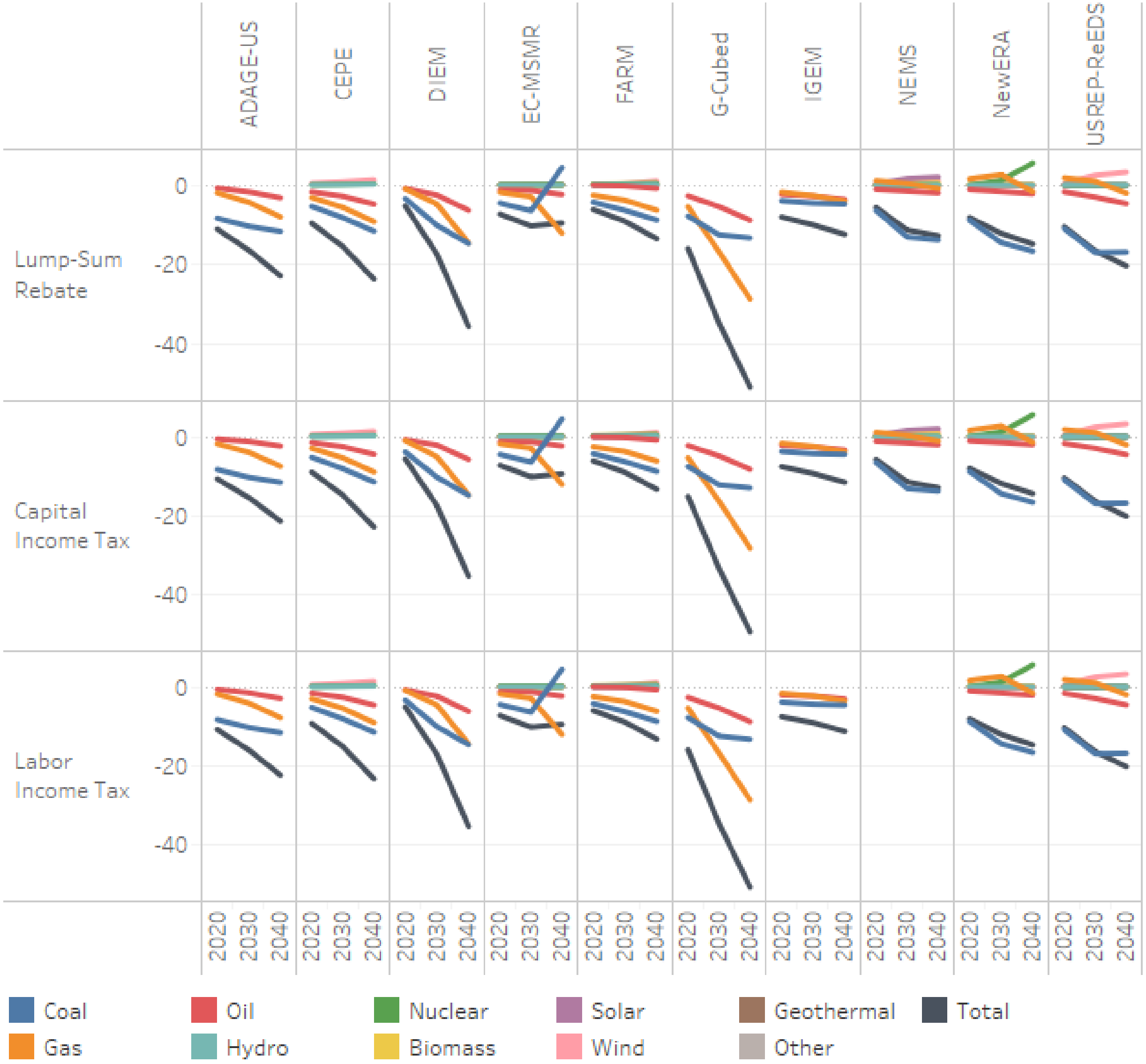

Figure S5: Change in Primary Energy Demand from Reference (Exajoules per Year).

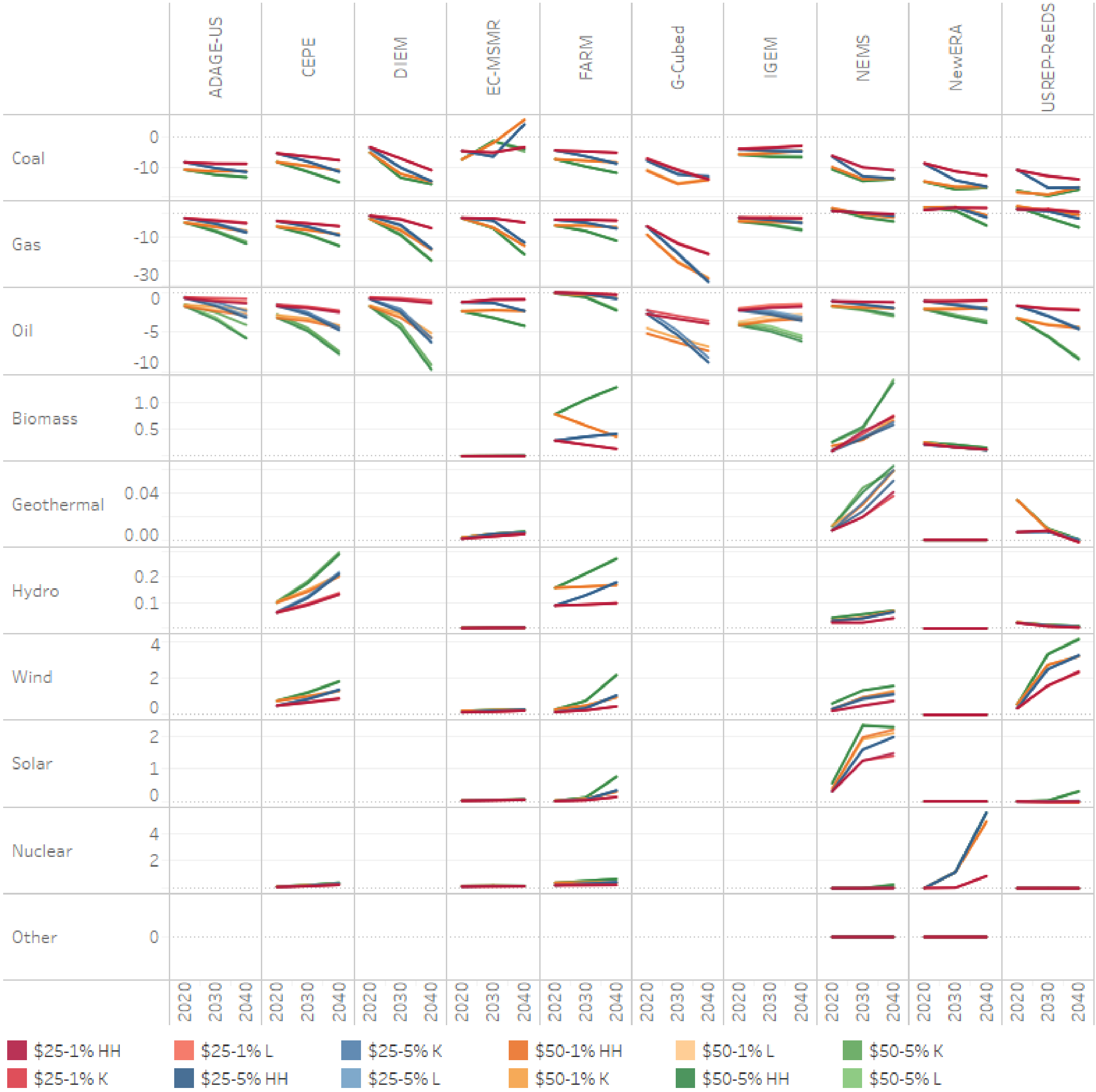

Figure S6: Change in Primary Energy by Fuel from Reference (Exajoules per Year).

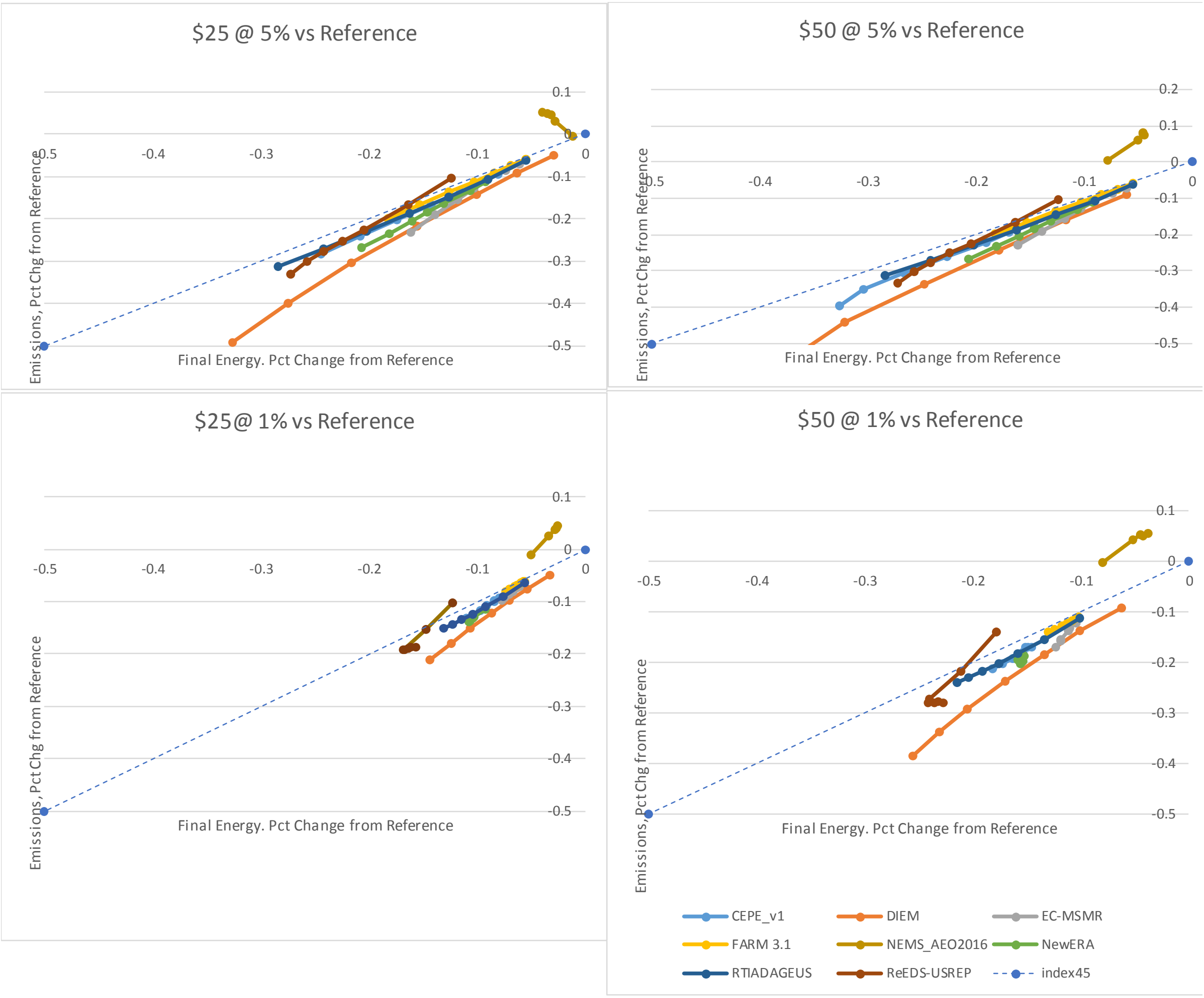

Notes: Graphs show final energy and emissions in percentage change from reference. The blue dotted line represents points where the percentage changes are equal. Generally, model results move chronologically from upper-right to lower-left as emissions and energy use decline.

Figure S7: Industrial Sector Emissions and Final Energy Use

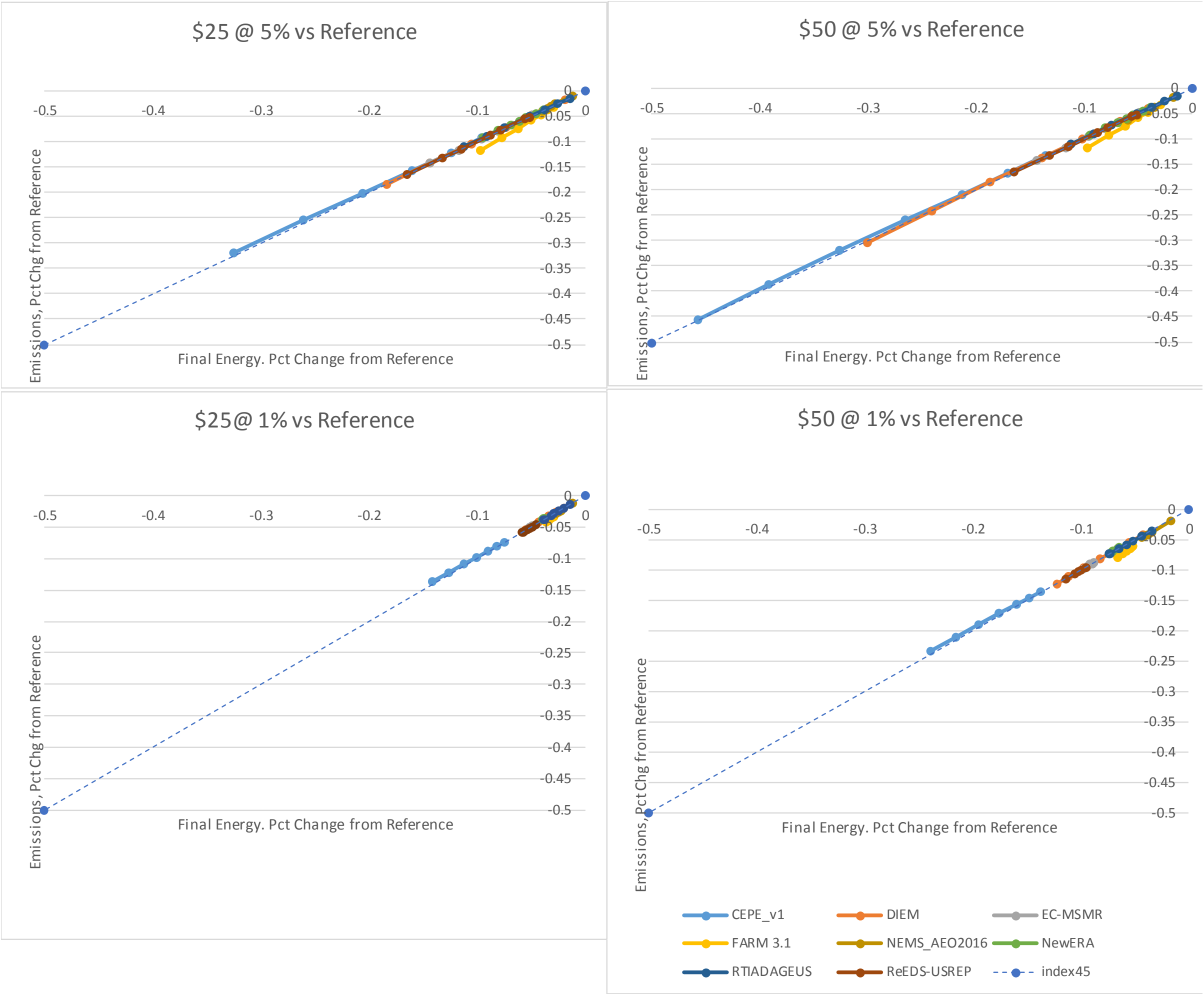

Notes: Graphs show final energy and emissions in percentage change from reference. The blue dotted line represents points where the percentage changes are equal. Generally, model results move chronologically from upper-right to lower-left as emissions and energy use decline.

Figure S8: Transportation Sector Emissions and Final Energy Use

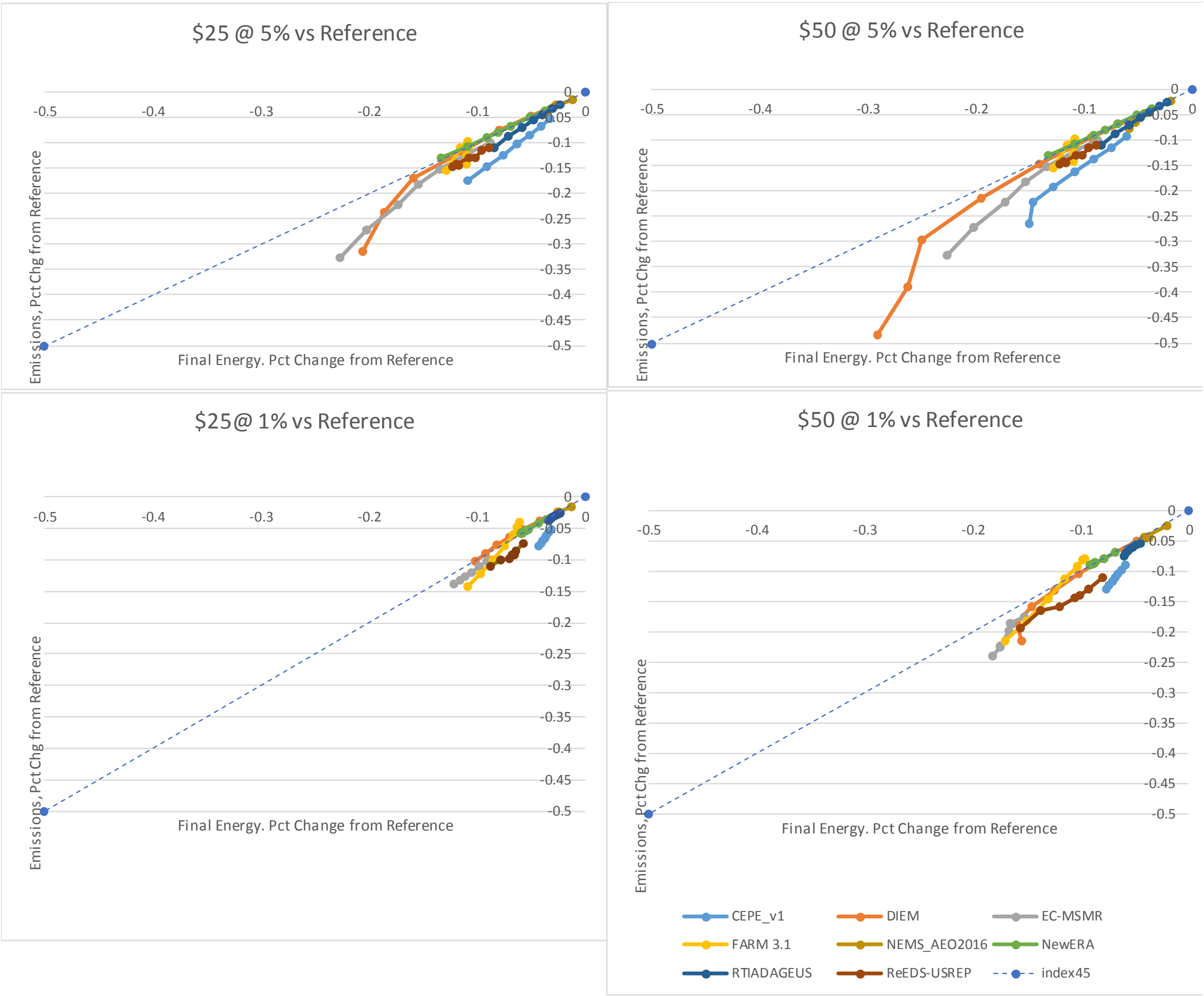

Notes: Notes: Graphs show final energy and emissions in percentage change from reference. The blue dotted line represents points where the percentage changes are equal. Generally, model results move chronologically from upper-right to lower-left as emissions and energy use decline.

Figure S9: Residential Sector Emissions and Final Energy Use.

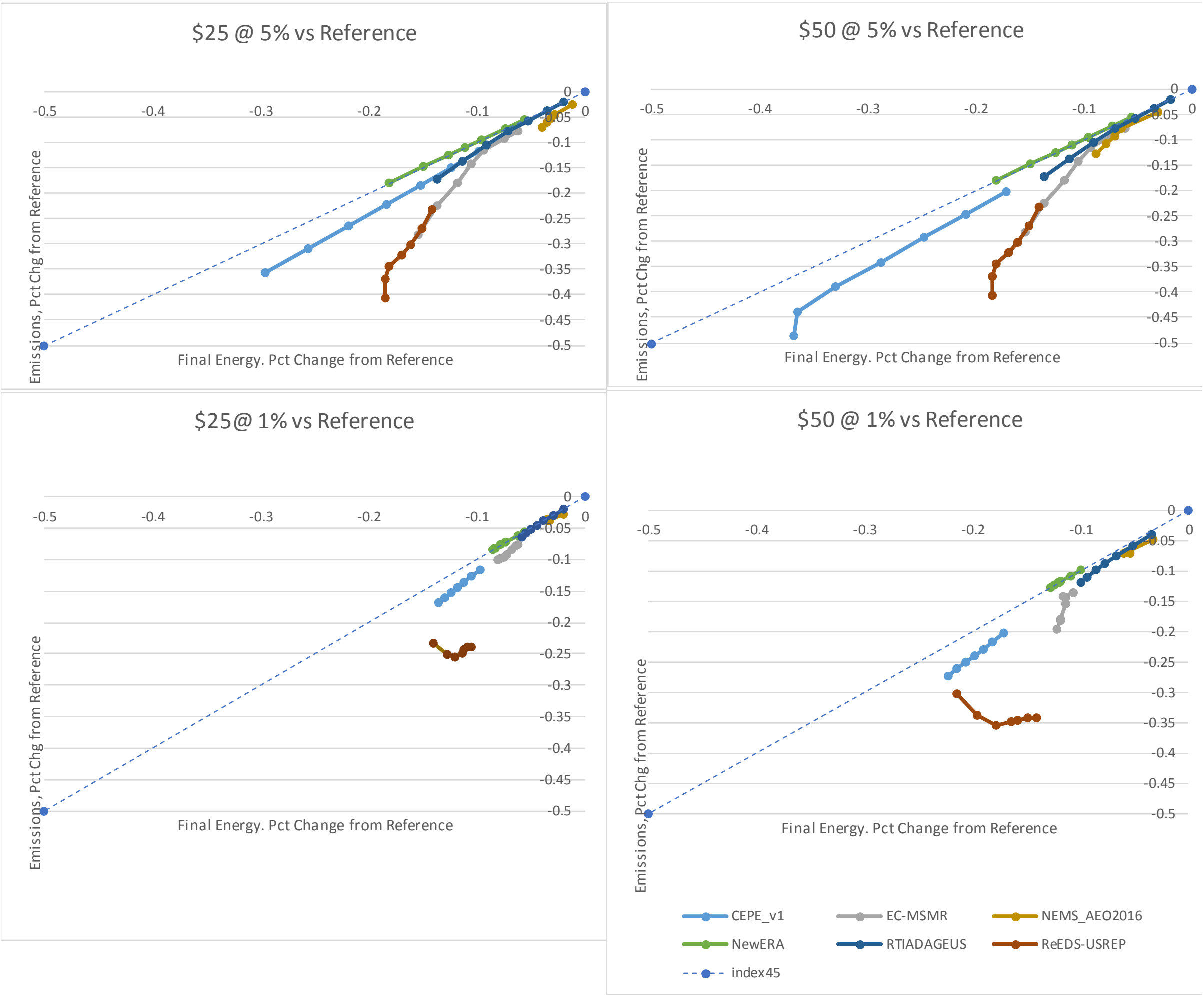

Notes: Graphs show final energy and emissions in percentage change from reference. The blue dotted line represents points where the percentage changes are equal. Generally, model results move chronologically from upper-right to lower-left as emissions and energy use decline.

Figure S10: Commercial Sector Emissions and Energy Use.

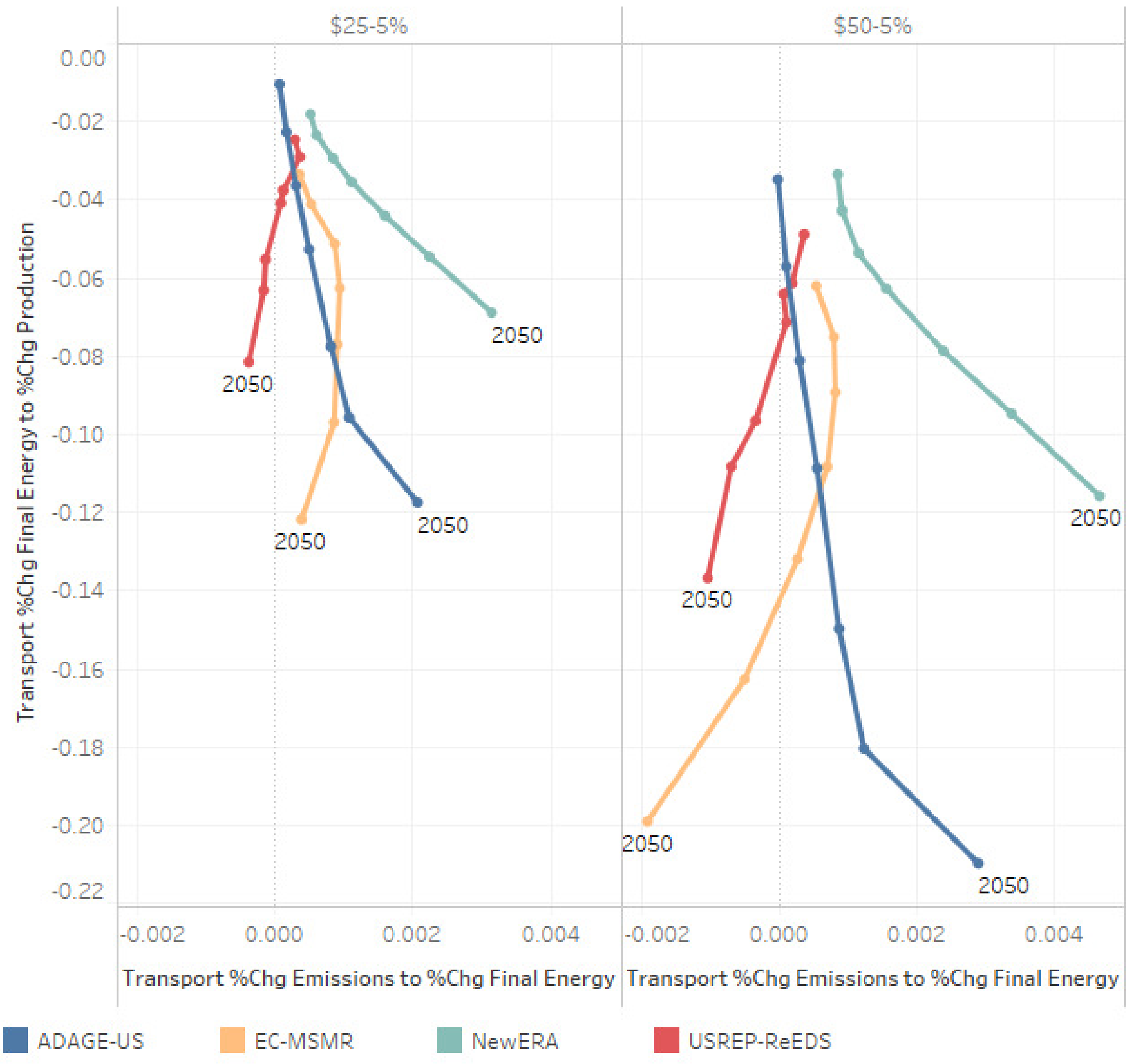

Figure S11: Kaya Decomposition of the Transportation Sector (2020-2050).

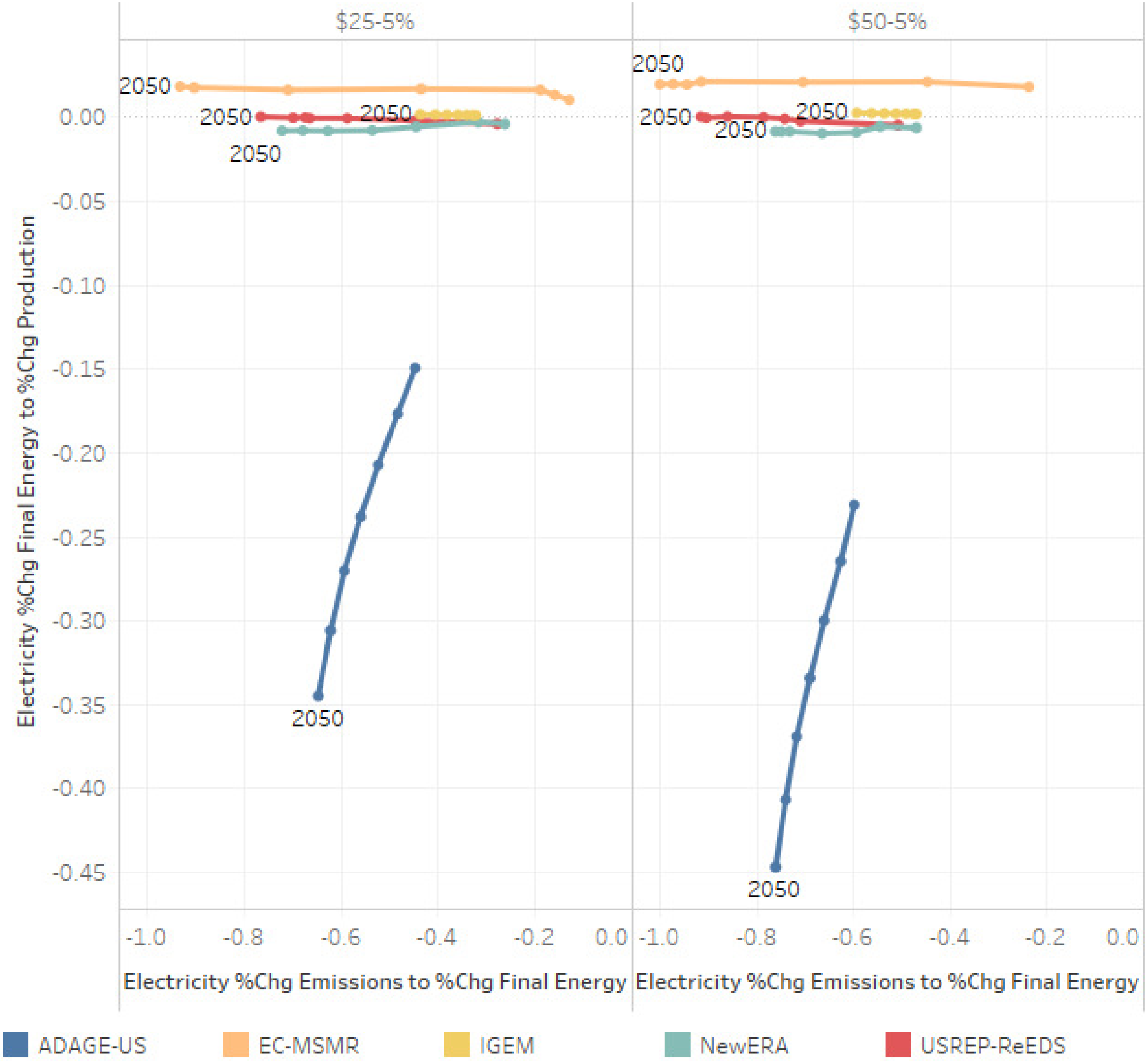

Figure S7: Kaya Decomposition of the Electricity Sector.

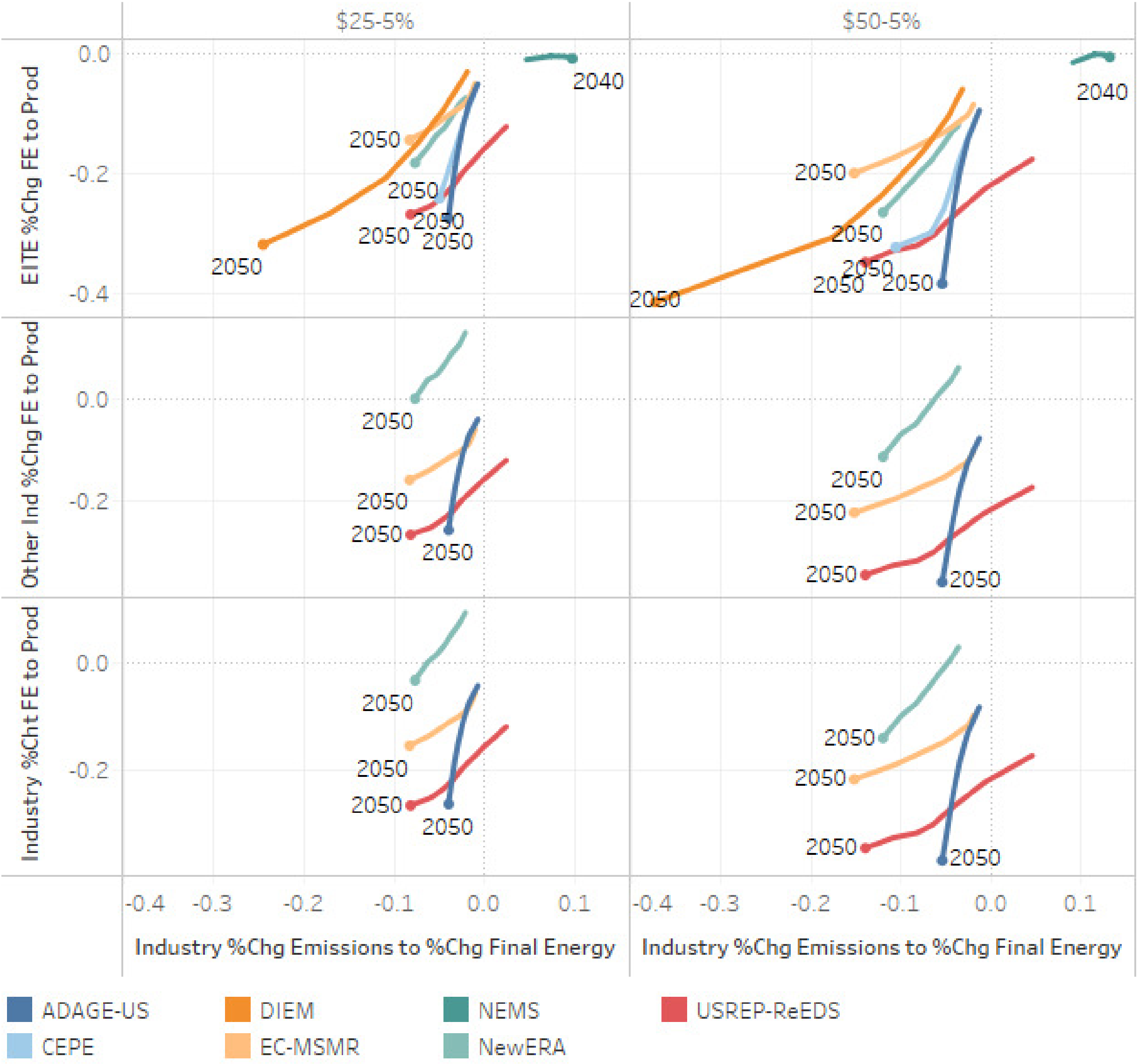

Figure S12: Kaya Decomposition of the Industrial Sector.

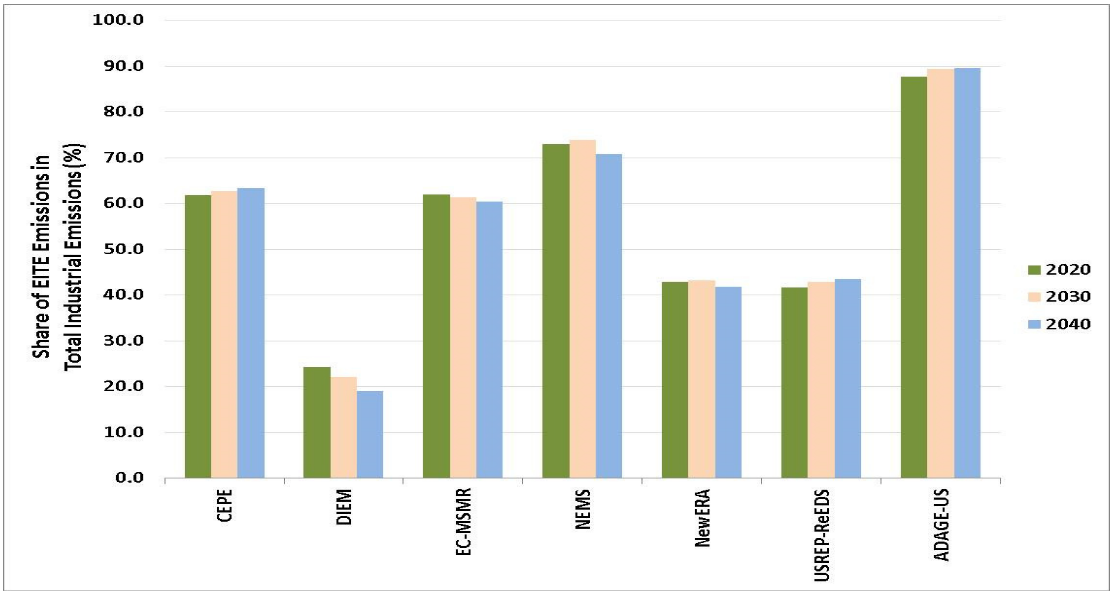

Figure S13: Share of EITE Emissions Relative to Total Industrial Emissions (%).

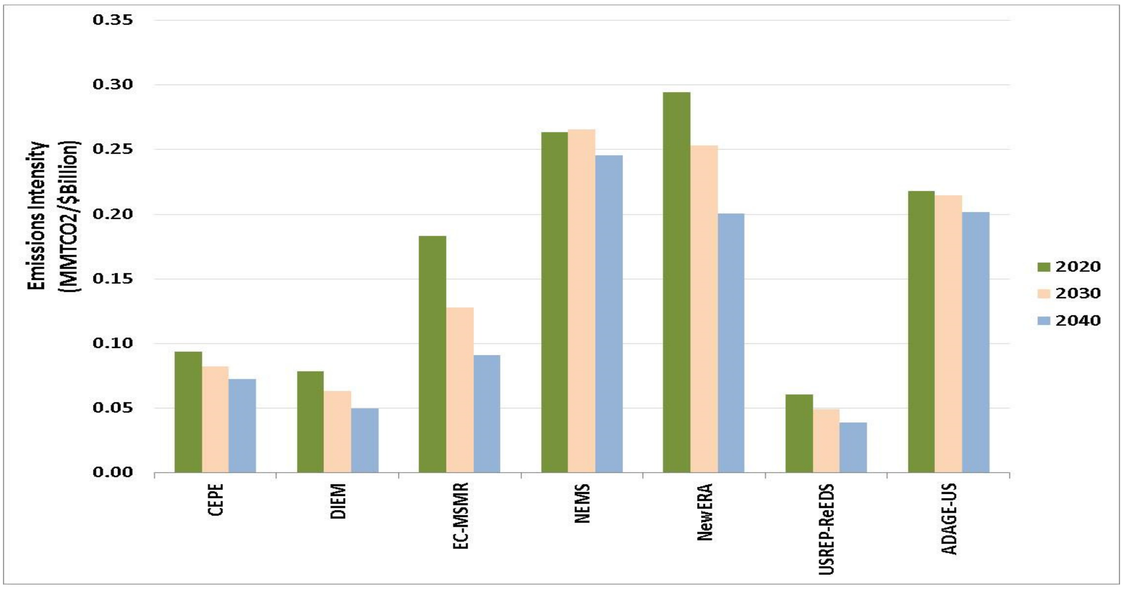

Figure S14: EITE Industries Emissions Intensity (MMTCO2 per $ billion).
